# Impact of treatment of COVID-19 with sotrovimab on post-acute sequelae of COVID-19 (PASC): an analysis of National COVID Cohort Collaborative (N3C) data

**DOI:** 10.1007/s15010-025-02505-z

**Published:** 2025-03-22

**Authors:** Myriam Drysdale, Rose Chang, Tracy Guo, Mei Sheng Duh, Jennifer Han, Helen Birch, Catherine Sharpe, Daisy Liu, Sarah Kalia, Melissa Van Dyke, Maral DerSarkissian, Iain A. Gillespie

**Affiliations:** 1 GSK, 79 New Oxford Street, London, WC1A 1DG UK; 2 Analysis Group, Inc., Boston, MA USA; 3 GSK, Collegeville, PA USA; 4 GSK, Stevenage, Hertfordshire UK; 5 Analysis Group, Inc., Los Angeles, CA USA

**Keywords:** COVID-19, Post-acute sequelae of COVID-19, Sotrovimab

## Abstract

**Purpose:**

To assess the impact of early sotrovimab treatment versus no treatment on the risk of developing post-acute sequelae of COVID-19 (PASC; long COVID) in patients (age ≥ 12 years) with COVID-19 at high risk for progression to severe disease.

**Methods:**

Retrospective cohort study using the US National COVID Cohort Collaborative (N3C) data. Phase 1 identified and assessed multiple definitions of PASC; Phase 2 evaluated the effectiveness of sotrovimab for reducing the risk of PASC, utilizing definitions from Phase 1. Average treatment effect in the treated (ATT)-weighted Cox proportional hazards regression models were used to compare time to event for PASC between high-risk patients who received sotrovimab treatment between May 26, 2021 and April 5, 2022, and high-risk patients with COVID-19 diagnosed between May 26, 2021 and March 26, 2022 who did not receive any treatment for COVID-19 during the acute phase or any pre-exposure prophylaxis against SARS-CoV-2.

**Results:**

A total of 9,504 sotrovimab-treated and 619,668 untreated patients were included in the main analysis. Most baseline characteristics were balanced between the two cohorts after ATT weighting. The doubly robust ATT-weighted hazard ratio (95% confidence interval) was 0.92 (0.89–0.96) (*p* < 0.001), indicating that sotrovimab use was associated with a significantly lower risk of PASC. Results remained consistent in sensitivity analyses.

**Conclusion:**

In patients at high risk for severe COVID-19, the benefits of early sotrovimab treatment may extend beyond the acute phase of COVID-19 and contribute to the prevention of PASC symptoms.

**Supplementary Information:**

The online version contains supplementary material available at 10.1007/s15010-025-02505-z.

## Background

Post-acute sequelae of COVID-19 (PASC), or long COVID, is characterized by persistent and long-term symptoms of coronavirus disease 2019 (COVID-19) that extend beyond the acute phase of the disease and can range widely in severity and presentation [1, 2]. As of March 2024, the Centers for Disease Control and Prevention estimates approximately 30% of adults in the United States (US) with a history of severe acute respiratory syndrome coronavirus 2 (SARS-CoV-2) infection reported having experienced PASC [3, 4].

PASC impacts physical, cognitive, and mental health, and affects multiple organ systems, resulting in increased healthcare resource use, impaired quality of life, and increased susceptibility to viral or bacterial infection [2, 5, 6]. Studies have suggested that over 200 symptoms may be attributed to PASC [7, 8], and long-term complications can occur in individuals with severe, mild, or asymptomatic SARS-CoV-2 infection [9]. Vaccination prior to infection has been shown to confer only partial protection in the post-acute phase of COVID-19 [7], emphasizing the need for other strategies for reducing the long-term consequences of COVID-19. Clinical recommendations for PASC focus on treating symptoms and underlying comorbidities, as there is currently no specific treatment for PASC [10, 11].

Sotrovimab is a dual-action monoclonal antibody (mAb) that neutralizes SARS-CoV-2 [12]. Despite data to indicate reduced in vitro neutralization activity against Omicron variants [13], real-world studies have shown continued effectiveness of sotrovimab in high-risk patients with mild-to-moderate COVID-19 during periods of Omicron BA.2 and BA.5 subvariant predominance [14, 15]. However, the impact of sotrovimab in preventing PASC and/or decreasing the symptom characteristics and severity of PASC has not yet been studied. This study evaluated the impact of early treatment with sotrovimab, compared with no treatment, on the risk of developing PASC in patients with COVID-19 at high risk for progression to severe disease.

## Methods

### Study design

This was a retrospective cohort study, conducted in two phases. Phase 1 aimed to identify and assess multiple definitions of PASC, based on a review of the literature (see the Supplementary Appendix for further details), and select one for use in Phase 2; Phase 2 leveraged these definitions in the evaluation of the effectiveness of sotrovimab for reducing the risk of PASC. Five PASC definitions were evaluated in Phase 1:

1. Use of the International Classification of Diseases, Tenth Revision, Clinical Modification (ICD-10-CM) diagnosis code U09.9 for long COVID at least 30 days after the first ICD-10 diagnosis code for COVID-19 (U07.1) or a positive polymerase chain reaction (PCR) or antigen test for COVID-19 in the observation period;
2. Referral to a long-COVID clinic at least once 30 days after diagnosis of COVID-19 in the observation period;
3. Demonstration of PASC (occurrence of at least one of the 26 symptoms defined by Bull-Otterson [1] and identified using ICD-10-CM codes) at least 30 days after the first ICD-10 diagnosis code for COVID-19 (U07.1) or a positive PCR or antigen test for COVID-19 in the observation period;
4. Demonstration of PASC (occurrence of at least one of the 26 symptoms defined by Bull-Otterson [1]) at least 60 days after the first ICD-10 diagnosis code for COVID-19 (U07.1) or a positive PCR or antigen test for COVID-19 in the observation period;
5. Demonstration of PASC (occurrence of at least one of the 26 symptoms defined by Bull-Otterson [1]) at least 90 days after the first ICD-10 diagnosis code for COVID-19 (U07.1) or a positive PCR or antigen test for COVID-19 in the observation period.

Pairwise overlap between the definitions was assessed, with a main definition and a sensitivity definition selected for use in Phase 2, based on the degree of overlap.

Patients eligible for Phase 2 were aged ≥ 12 years at index and had confirmed COVID-19. Patients who received treatment with mAbs, antivirals, or tixagevimab/cilgavimab during the baseline period, or were re-infected with COVID-19, were excluded. Full inclusion and exclusion criteria are shown in Supplementary Table S1.

**Fig. 1:**
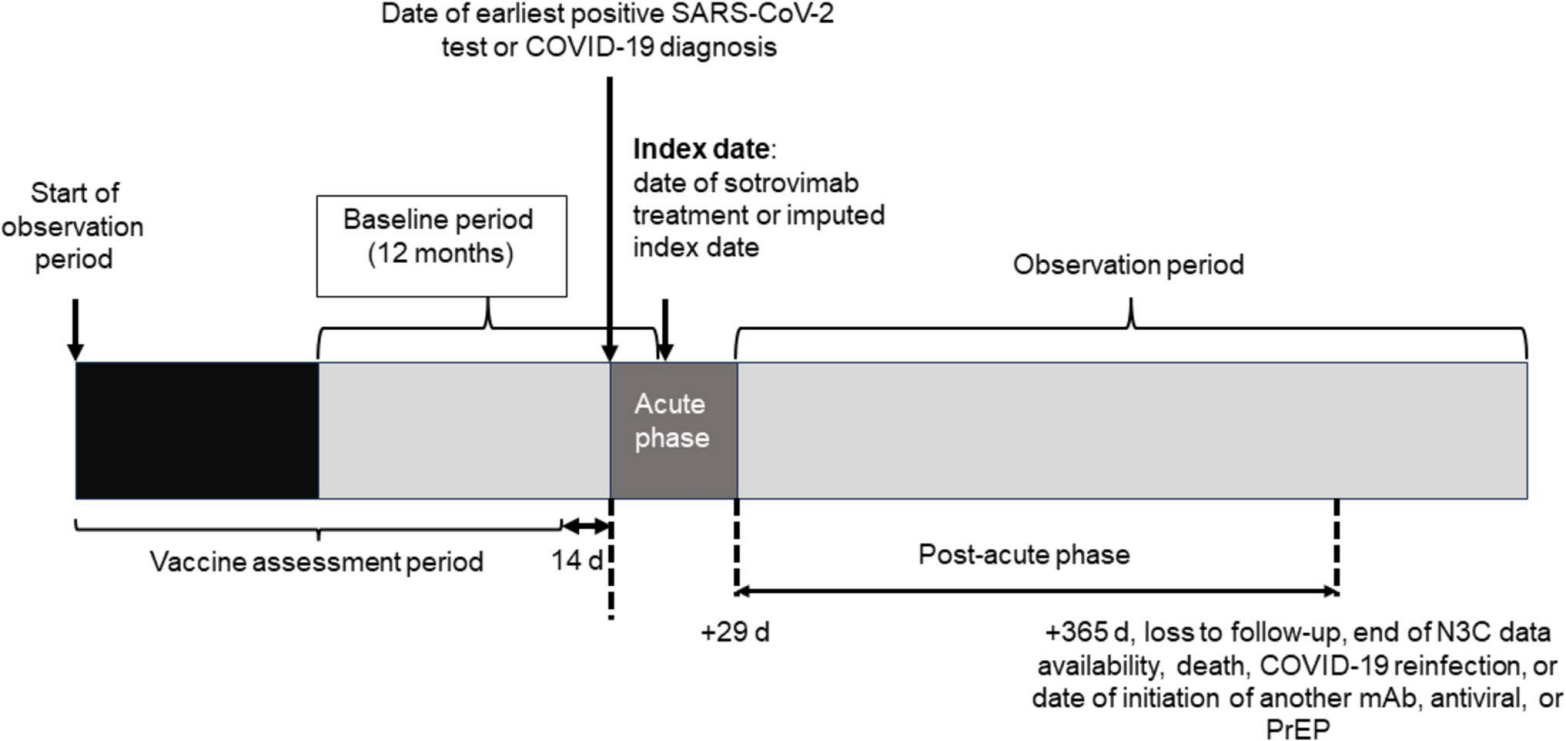
Study (Phase 2) design. *COVID-19* coronavirus disease 2019, *d* days, *mAb* monoclonal antibody, *N3C *National COVID Cohort Collaborative, *PrEP* pre-exposure prophylaxis, *SARS-CoV-2* severe acute respiratory syndrome coronavirus 2

The design of Phase 2 is illustrated in Fig. 1. For the main Phase 2 analyses, two cohorts of patients were defined (see Table 1 for full criteria). The first cohort included high-risk patients (i.e., meeting the emergency use authorization [EUA] criteria for sotrovimab) with COVID-19 who received treatment with sotrovimab between May 26, 2021 and April 5, 2022, with their index date being the date of sotrovimab infusion. The index date for the second cohort – untreated, high-risk patients with a COVID-19 diagnosis between May 26, 2021 and March 26, 2022 – was randomly selected, based on the distribution of the time from positive SARS-CoV-2 test to sotrovimab infusion date for the treated cohort (range 1–10 days; mean [standard deviation] 1.3 [1.5] days).

**Table 1:** Study cohorts

Cohort	Description
Sotrovimab-treated, high-risk	• High-risk patients (i.e., meeting the EUA criteria for sotrovimab^a^) with COVID-19 who received treatment with sotrovimab between May 26, 2021 and April 5, 2022 in an outpatient or ER setting within 10 days of COVID-19 diagnosis^b^ • Not hospitalized between the date of COVID-19 diagnosis and the index date • Did not receive any other treatment (i.e., mAb or antiviral) for COVID-19 or for pre-exposure prophylaxis against SARS-CoV-2 in any setting during the acute phase
Untreated, high-risk	• High-risk patients with COVID-19 diagnosed between May 26, 2021 and March 26, 2022 who did not receive any treatment (mAb or antiviral) for COVID-19 or for pre-exposure prophylaxis against SARS-CoV-2 in any setting during the acute phase • Not hospitalized between the date of COVID-19 diagnosis and the index date
Untreated, high-risk	• A random sample of 25% of patients who met the following criteria: high-risk with COVID-19 diagnosed after March 1, 2020 who did not receive any treatment (i.e., mAb or antiviral) for COVID-19 or for pre-exposure prophylaxis against SARS-CoV-2 in any setting during the acute phase
Untreated, non-high-risk	• A random sample of 25% of patients who met the following criteria: non-high-risk with COVID-19 who did not receive any treatment (i.e., mAb or antiviral) for COVID-19 or for pre-exposure prophylaxis against SARS-CoV-2 in any setting during the acute phase

^a^Includes age ≥ 65 years, obesity, pregnancy, history of CKD, history of diabetes type 1 or type 2, immunosuppressive disease, immunosuppressive treatment, cardiovascular disease (including congenital heart disease) or hypertension, chronic lung disease (i.e., COPD, asthma, interstitial lung disease, cystic fibrosis, pulmonary hypertension), sickle cell disease, neurodevelopmental disorders, medical-related technological dependence, liver disease, anti-diabetic therapies, acute or acute-on-chronic respiratory failure, pulmonary hypertension, heart failure, acquired heart disease, non-asthma/COPD chronic respiratory diseases, ICS-containing therapies

^b^Positive PCR or antigen test for SARS-CoV-2 or first date of diagnosis of U07.1 (COVID-19, virus identified)

*CKD* chronic kidney disease, *COPD* chronic obstructive pulmonary disease, *COVID-19* coronavirus disease 2019, *ER* emergency room, *EUA* emergency use authorization, *ICS* inhaled corticosteroid, *mAb* monoclonal antibody, *PCR* polymerase chain reaction, *SARS-CoV-2* severe acute respiratory syndrome coronavirus 2

Two further cohorts were defined in Phase 2: untreated, high-risk patients with a COVID-19 diagnosis after March 1, 2020; and untreated, non-high-risk patients. For both cohorts, the index date was the earliest COVID-19 diagnosis date between March 1, 2020 and June 15, 2023.

Baseline for all cohorts was defined as the 12 months prior to the index date, during which demographic and clinical characteristics were described. All available data (up to 14 days prior to COVID-19 diagnosis date) were used to assess vaccination status. The acute phase was defined as the time from COVID-19 diagnosis until 29 days following diagnosis date, during which time treatments for COVID-19 were identified. The observation period was defined from day 30 following diagnosis until the earliest of the following: day 365 following diagnosis date, loss to follow-up (date of end of clinical activity in the electronic health record), end of National COVID Cohort Collaborative (N3C) data availability (i.e., June 15, 2023), date of death, COVID-19 re-infection, or initiation of another mAb, antiviral, or tixagevimab/cilgavimab.

### Data source

The study utilized the US National Center for Advancing Translational Sciences (NCATS) N3C database. The N3C database is a centralized, secure, national clinical data resource created by the National Institute of Health (NIH), which aimed to aggregate and harmonize electronic health records data across clinical organizations in the US to rapidly advance the understanding of SARS-CoV-2 [16]. The N3C database includes clinical data for patients who represent diverse populations with respect to geography, socioeconomic status, race/ethnicity, age, and underlying medical conditions. A variety of encounter types (e.g., inpatient, outpatient, and emergency room [ER] visits) are represented in the N3C data. The N3C COVID-19 cohort consists of patients at participating sites with: (1) a positive a priori-defined SARS-CoV-2 laboratory test including a positive PCR test or antigen test; (2) a strong positive diagnosis code (i.e., diagnosis codes for coronavirus or COVID-19); or (3) two weak positive diagnosis codes during the same encounter or on the same day (i.e., diagnosis codes related to symptoms associated with COVID-19) before May 1, 2020 [17]. A project-specific Data Use Request (DUR) was submitted, and Institutional Review Board (IRB) approval was obtained prior to accessing N3C data.

### Statistical analysis

#### Phase 1

Descriptive analyses for five definitions of PASC were conducted for all study cohorts. Patients in each cohort meeting the definitions of PASC were summarized using absolute and relative frequencies. The overlap in patients meeting two or more PASC definitions was assessed, as a definition with high degrees of overlap with other definitions may be an indication of higher sensitivity (i.e., ability to identify true PASC diagnosis). Based on findings from the descriptive analyses, one main definition and one sensitivity definition of PASC was used in Phase 2.

#### Phase 2

Demographics and clinical characteristics of the cohorts were first described. Categorical variables were summarized as frequencies and proportions. Continuous variables were summarized as mean and standard deviation, and median and interquartile range (IQR). Additionally, the number and proportion of missing data were reported for each baseline characteristic.

The main analysis in Phase 2 assessed the risk of PASC in sotrovimab-treated and untreated high-risk patients. Average treatment effect in the treated (ATT) weighting, calculated from propensity scores (PS), was used to control for confounding by baseline characteristics and reduce non-comparability between cohorts. ATT-weighted Cox proportional hazards regression models were used to compare time to event for PASC between sotrovimab-treated versus untreated patients. Each patient was weighted by the ratio of the PS – defined as the probability of being high risk given an observed set of relevant baseline covariates – to the inverse of the PS. The distribution of patient characteristics was compared between the weighted cohorts using standardized differences to ensure balance. A standardized difference > 10% was considered to indicate meaningful imbalance [18]. Covariates that were imbalanced at baseline after weighting (standardized difference > 10%) and/or clinically relevant covariates were included in the Cox proportional hazards regression model in a doubly robust approach. Crude and doubly robust weighted hazard ratios (HRs) with 95% confidence intervals (CIs; presented in square parentheses) and *p*-values were generated. The main analysis was also conducted in subgroups of interest (adults aged ≥ 18 years, hospitalization and/or intensive care unit [ICU] admission during the acute phase, highest risk).

#### Sensitivity analyses

A multivariable Cox proportional hazards regression model adjusted for relevant baseline patient and clinical characteristics was used to evaluate the association between individual baseline characteristics and PASC. This served as a sensitivity analysis for the doubly robust, ATT-weighted analysis by using a different method to control for measured confounding and allowed for identification of predictors of PASC independent of treatment. A sensitivity analysis using administrative censoring was also conducted, in which patients were censored at day 365 following the diagnosis date, or at death. This follows a treatment policy strategy, where patients who have intercurrent events (e.g., COVID re-infection, initiation of another mAb, antiviral, or tixagevimab/cilgavimab) during the observation period would continue to be followed until the earliest of death or 365 days after diagnosis. The same ATT weighting method and modeling approach as described above were applied. A sensitivity analysis using a more stringent definition of PASC (based on the use of ICD-10 diagnosis codes or positive COVID-19 test and/or at least one visit to a PASC specialty clinic) was also conducted.

#### Untreated high-risk versus untreated non-high-risk patients

In order to evaluate whether being high risk in itself increased the risk of PASC, untreated high-risk patients were compared with untreated non-high-risk patients. As some baseline variables (e.g., age, comorbidities) used to balance cohorts were also components of the high-risk definition, however, the PS model for computing weights for these comparisons included a limited subset of covariates to avoid overadjustment.

## Results

A total of 2,824,136 patients with confirmed COVID-19 were identified between May 26, 2021 and March 26, 2022 (Supplementary Fig. S1). After applying the eligibility criteria, 9,504 sotrovimab-treated and 619,668 untreated patients were included in the main analysis.

### Phase 1–endpoint definition selection

Pairwise overlap in patients meeting definitions 3 to 5 was > 90% for all cohorts (Table 2). Across all cohorts, the overlap between definition 3 and definitions 1 and 2 was higher than that between both definition 4 and definition 5 and definitions 1 and 2. Given its high degree of overlap with all other definitions**,** PASC definition 3 was used as the main PASC definition in Phase 2 of the study. A composite of definition 1 and/or 2 was chosen for the sensitivity analysis assessing the effect of an alternative (more specific) PASC definition on the findings.

**Table 2:**
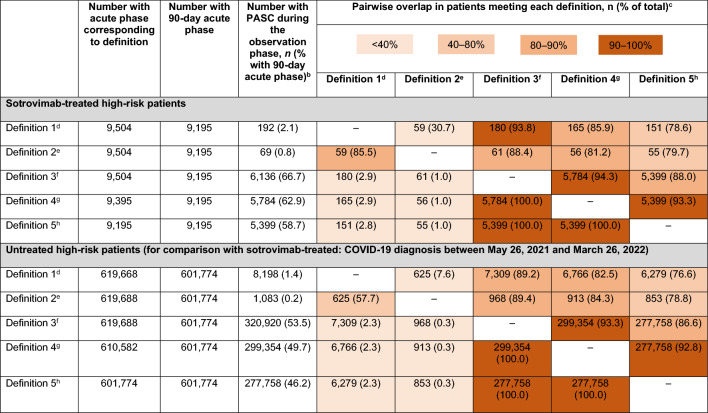
Overlap between definitions of PASC in the 90-day acute-phase sample^a^

^a^Cohort sample size depends on acute-phase length, which varies across PASC definitions (i.e., 30, 60, and 90 days); however, the same population should be used to assess overlap between definitions such that the percentages could be directly compared with each other. Since patients included in the 30-day and 60-day acute-phase samples would also be included in the 90-day acute-phase sample, the 90-day acute-phase sample was used for this analysis

^b^The observation period was defined from the end of the acute phase until the earliest of day 365 following the diagnosis date, loss to follow-up, end of N3C data availability, death, COVID-19 re-infection (second positive SARS-CoV-2 PCR/antigen test at least 45 days after the first positive SARS-CoV-2 PCR/antigen test or diagnosis of COVID-19, and after ≥ 1 negative SARS-CoV-2 PCR/antigen test), or initiation of another mAb, antiviral, or pre-exposure prophylaxis

^c^The pairwise overlap percentage is based on row total (i.e., the number of patients meeting the PASC definition in each column divided by the total number of patients meeting the PASC definition in each row)

^d^Definition 1 was defined as the use of U09.9 on or after October 1, 2021, or the use of B94.8 prior to October 1, 2021 at least 30 days after the first ICD-10 diagnosis code for COVID-19 (U07.1) or positive PCR or antigen test in the observation period

^e^Definition 2 was defined as at least one visit to a long-COVID specialty clinic 30 days after the first ICD-10 diagnosis code for COVID-19 (U07.1) or positive PCR or antigen test in the observation period

^f^Definition 3 was defined as demonstration of at least one of the 26 PASC symptoms (described in [1]) at least 30 days after the first ICD-10 diagnosis code for COVID-19 (U07.1) or positive PCR or antigen test in the observation period

^g^Definition 4 was defined as demonstration of at least one of the 26 PASC symptoms (described in [1]) at least 60 days after the first ICD-10 diagnosis code for COVID-19 (U07.1) or positive PCR or antigen test in the observation period

^h^Definition 5 was defined as demonstration of at least one of the 26 PASC symptoms (described in [1]) at least 90 days after the first ICD-10 diagnosis code for COVID-19 (U07.1) or positive PCR or antigen test in the observation period

*COVID-19* coronavirus disease 2019, *ICD-10* International Classification of Diseases, Tenth Revision, *mAb* monoclonal antibody, *N3C *National COVID Cohort Collaborative, *PASC *post-acute sequelae of COVID-19, *PCR* polymerase chain reaction, *SARS-CoV-2* severe acute respiratory syndrome coronavirus 2

### Baseline characteristics

Baseline characteristics of treated (*n* = 9,504) and untreated (*n* = 619,668) patients are shown in Table 3. Sotrovimab-treated high-risk patients were, on average, older than untreated high-risk patients (mean 60 vs 53 years, respectively), and more often White, obese, and smokers. Treated patients also had a higher comorbid burden, as evidenced by the mean Charlson Comorbidity Index (CCI), and a higher proportion receiving concomitant medication during the baseline and acute-phase periods than untreated patients. The most common EUA criteria were cardiovascular disease or hypertension (59.5% and 46.2% among treated and untreated patients, respectively), and age at index date > 65 years (47.5% treated and 32.9% untreated). The median (IQR) number of EUA criteria was 3.0 (1.0, 4.0) for treated patients and 2.0 (1.0, 3.0) for untreated patients. The percentage of treated patients with healthcare encounters during the acute phase was also higher than for untreated patients.

**Table 3:** Baseline characteristics of high-risk sotrovimab-treated versus high-risk untreated patients prior to and after ATT weighting

	Unweighted sample			Weighted sample^a^		
	High-risk sotrovimab-treated (*n* = 9,504)	High-risk untreated (*n* = 619,668)	Std. diff. (%)	High-risk sotrovimab-treated (*n* = 9,504)	High-risk untreated (*n* = 9,523)	Std. diff. (%)
**Demographic characteristics**						
Age at index (years)						
12–17, *n* (%)	53 (0.6)	23,397 (3.8)	22.2*	53 (0.6)	91 (1.0)	5.0
18–64, *n* (%)	4,938 (52.0)	392,671 (63.4)	23.3*	4,938 (52.0)	5,045 (53.0)	2.0
≥ 65, *n* (%)	4,513 (47.5)	203,600 (32.9)	30.2*	4,513 (47.5)	4,383 (46.0)	2.9
Mean (SD)	60.2 (17.3)	53.0 (19.2)	39.2*	60.2 (17.3)	60.3 (17.2)	0.9
Median (IQR)	63.8 (48.0, 73.0)	54.8 (37.7, 68.2)	–	63.8 (48.0, 73.0)	63.0 (49.0, 73.0)	–
Sex, *n* (%)						
Female	5,648 (59.4)	386,015 (62.3)	5.9	5,648 (59.4)	5,644 (59.3)	0.3
Male	3,855 (40.6)	233,565 (37.7)	5.9	3,855 (40.6)	3,878 (40.7)	0.3
Other/unknown	1 (0.0)	88 (0.0)	0.3	1 (0.0)	1 (0.0)	0.0
Ethnicity​, *n* (%)						
Non-Hispanic or non-Latino	8,826 (92.9)	515,603 (83.2)	30.1*	8,826 (92.9)	8,843 (92.9)	0.0
Hispanic or Latino	483 (5.1)	52,957 (8.5)	13.8*	483 (5.1)	483 (5.1)	0.0
Other/unknown	195 (2.1)	51,108 (8.2)	28.3*	195 (2.1)	197 (2.1)	0.1
Race, *n* (%)						
White	8,175 (86.0)	464,156 (74.9)	28.3*	8,175 (86.0)	8,193 (86.0)	0.0
Black or African American	819 (8.6)	92,848 (15.0)	19.8*	819 (8.6)	819 (8.6)	0.1
Asian or Pacific Islander	165 (1.7)	15,358 (2.5)	5.2	165 (1.7)	165 (1.7)	0.0
Other/unknown^b^	345 (3.6)	47,306 (7.6)	17.4*	345 (3.6)	346 (3.6)	0.0
US geographic region, *n* (%)						
West	1,631 (17.2)	58,335 (9.4)	23.0*	1,631 (17.2)	1,649 (17.3)	0.4
Midwest	3,771 (39.7)	269,716 (43.5)	7.8	3,771 (39.7)	3,750 (39.4)	0.6
South	3,081 (32.4)	130,644 (21.1)	25.8*	3,081 (32.4)	3,092 (32.5)	0.1
Northeast	214 (2.3)	42,059 (6.8)	22.0*	214 (2.3)	216 (2.3)	0.1
Other/unknown	807 (8.5)	118,914 (19.2)	31.4*	807 (8.5)	816 (8.6)	0.3
BMI category (kg/m^2^),^c^ *n* (%)						
< 18.5 (underweight)	40 (0.4)	2,634 (0.4)	0.1	40 (0.4)	40 (0.4)	0.1
18.5–24.9 (normal)	1,442 (15.2)	62,276 (10.0)	15.5*	1,442 (15.2)	1,452 (15.2)	0.2
25.0–29.9 (overweight)	2,219 (23.3)	84,233 (13.6)	25.3*	2,219 (23.3)	2,225 (23.4)	0.0
≥ 30 (obese)	3,742 (39.4)	156,387 (25.2)	30.6*	3,742 (39.4)	3,732 (39.2)	0.4
Unknown	2,061 (21.7)	314,138 (50.7)	63.3*	2,061 (21.7)	2,074 (21.8)	0.2
**Clinical characteristics**						
EUA criteria, *n* (%)^d,e^						
Cardiovascular disease (including congenital heart disease) or hypertension	5,652 (59.5)	286,237 (46.2)	26.8*	5,652 (59.5)	5,675 (59.6)	0.3
Age at index date ≥ 65 years	4,513 (47.5)	203,600 (32.9)	30.2*	4,513 (47.5)	4,383 (46.0)	2.9
Immunosuppressive disease^f^	3,876 (40.8)	123,357 (19.9)	46.6*	3,876 (40.8)	3,882 (40.8)	0.0
Acquired heart disease	3,315 (34.9)	126,636 (20.4)	32.7*	3,315 (34.9)	3,392 (35.6)	1.5
History of diabetes type 2	2,436 (25.6)	113,291 (18.3)	17.8*	2,436 (25.6)	2,439 (25.6)	0.0
Chronic lung disease (COPD or asthma)	1,971 (20.7)	112,467 (18.1)	6.5	1,971 (20.7)	2,232 (23.4)	6.5
Obesity^g^	4,091 (43.0)	189,211 (30.5)	26.2*	4,091 (43.0)	4,114 (43.2)	0.3
History of CKD (any stage)	1,474 (15.5)	35,629 (5.7)	32.1*	1,474 (15.5)	1,508 (15.8)	0.9
Non-asthma and non-COPD chronic respiratory diseases	1,170 (12.3)	46,645 (7.5)	16.1*	1,170 (12.3)	1,176 (12.4)	0.1
Heart failure	858 (9.0)	26,800 (4.3)	18.9*	858 (0.9)	873 (9.2)	0.5
Liver disease	837 (8.8)	35,572 (5.7)	11.8*	837 (8.8)	839 (8.8)	0.0
Pregnancy	580 (6.1)	41,591 (6.7)	2.5	580 (6.1)	367 (3.8)	10.4*
History of CKD (stage ≥ 3)	570 (6.0)	12,769 (2.1)	20.1*	570 (6.0)	504 (5.3)	3.0
Neurodevelopmental disorders	378 (4.0)	40,615 (6.6)	11.6*	378 (4.0)	374 (3.9)	0.3
Pulmonary hypertension	337 (3.5)	7,118 (1.1)	15.9*	337 (3.5)	343 (3.6)	0.3
Immunosuppressive treatment^h^	333 (2.5)	16,921 (2.7)	4.4	333 (3.5)	298 (3.1)	2.1
Anti-diabetic therapies	230 (2.4)	9,736 (1.6)	6.1	230 (2.4)	208 (2.2)	1.5
History of diabetes type 1	220 (2.3)	8,349 (1.3)	7.2	220 (2.3)	221 (2.3)	0.1
Acute or acute-on-chronic respiratory failure	208 (2.2)	9,840 (1.6)	4.4	208 (2.2)	321 (3.4)	7.2
A medical-related technological dependence^i^	186 (2.0)	4,379 (0.7)	10.9*	186 (2.0)	194 (2.0)	0.5
ICS-containing therapies	145 (1.5)	8,735 (1.4)	1.0	145 (1.5)	186 (2.0)	3.3
Sickle cell disease	21 (0.2)	2,371 (0.4)	2.9	21 (0.2)	24 (0.2)	0.5
Number of EUA criteria						
Mean (SD)	3.3 (2.2)	2.2 (1.6)	55.5*	3.3 (2.2)	3.3 (2.2)	0.2
Median (IQR)	3.0 (1.0, 4.0)	2.0 (1.0, 3.0)	–	3.0 (1.0, 4.0)	3.0 (1.0, 4.0)	–
Current/former smoker, *n* (%)	1,791 (18.8)	47,598 (7.7)	33.4*	1,791 (18.8)	1,810 (19.0)	0.4
CCI, mean (SD)	1.7 (2.3)	0.9 (1.5)	44.6*	1.7 (2.3)	1.7 (2.3)	0.3
Received any concomitant medication, *n* (%)						
During baseline	9,093 (95.7)	517,588 (83.5)	40.6*	9,093 (95.7)	9,111 (95.7)	0.0
During acute phase	8,196 (86.2)	230,029 (37.1)	117.1*	8,196 (86.2)	8,128 (86.3)	0.2
During acute phase before index	6,565 (69.1)	33,782 (5.5)	174.7*	6,565 (69.1)	1,126 (11.8)	143.6*
Healthcare encounters during baseline^j^						
*n* (%)	9,395 (98.9)	601,757 (97.1)	12.4*	9,395 (98.9)	9,440 (99.1)	2.8
Mean (SD)	36.6 (36.2)	19.4 (21.4)	57.9*	36.6 (36.2)	36.8 (38.2)	0.4
Median (IQR)	26.0 (12.0, 49.0)	13.0 (6.0, 25.0)	–	26.0 (12.0, 49.0)	25 (12.0, 48.0)	–
Healthcare encounters during acute phase^j^						
*n* (%)	8,697 (91.5)	417,525 (67.4)	62.6*	8,697 (91.5)	8,463 (88.9)	8.9
Mean (SD)	4.6 (4.3)	2.1 (2.8)	66.8*	4.6 (4.3)	4.6 (4.7)	1.0
Median (IQR)	3.0 (1.0, 6.0)	1.0 (0.0, 3.0)	–	3.0 (1.0, 6.0)	3.0 (1.0, 6.0)	–
Healthcare encounters during acute phase^j^ before index						
*n* (%)	6,531 (68.7)	99,547 (16.1)	125.9*	6,531 (68.7)	2,436 (25.6)	95.8*
Mean (SD)	1.1 (1.0)	0.2 (0.5)	104.3*	1.1 (1.0)	0.4 (0.8)	76.6*
Median (IQR)	1.0 (0.0, 1.0)	0.0 (0.0, 0.0)	–	1.0 (0.0, 1.0)	0.0 (0.0, 0.0)	–
**COVID-19 disease history**						
Vaccination status, *n* (%)^e,k^						
Fully vaccinated with booster^l^	1,594 (16.8)	142,339 (23.0)	15.6*	1,594 (16.8)	1,824 (19.2)	6.2
Full vaccinated without booster^m^	1,505 (15.8)	52,349 (8.4)	22.8*	1,505 (15.8)	1,245 (13.1)	7.9
Partially vaccinated^n^	333 (3.5)	15,933 (2.6)	5.4	333 (3.5)	375 (3.9)	2.3
No record of vaccination	6,072 (63.9)	409,047 (66.0)	4.4	6,072 (63.9)	6,079 (63.8)	0.1
Time from last COVID-19 vaccination dose to index (months), *n*						
*n*	3,432	210,621		3,432	3,444	
Mean (SD)	6.2 (3.3)	6.0 (3.2)	7.6	6.2 (3.3)	6.0 (3.4)	6.0
Median (IQR)	5.3 (3.5, 9.3)	6.0 (3.3, 8.5)	–	5.3 (3.5, 9.3)	5.0 (3.0, 9.0)	–
Test used to diagnose COVID-19, *n* (%)						
PCR	4,230 (44.5)	339,476 (54.8)	20.7*	4,230 (44.5)	4,197 (44.1)	0.9
Other/unknown^o^	5,274 (55.5)	280,192 (45.2)	–	5,274 (55.5)	5,326 (55.9)	–
COVID-19 diagnosis in ER, *n* (%)	1,011 (10.6)	89,929 (14.5)	11.7*	1,011 (10.6)	1,022 (10.7)	0.3

^*^Indicates potential confounder (standardized difference > 10%)

^a^Covariates included in the propensity score used to generate ATT weights were those with standardized difference > 10% before weighting and/or with clinical importance as potential confounders, and included: age at index date, sex, race, ethnicity, US geographic region, BMI, EUA criteria (cardiovascular disease or hypertension, immunosuppressive disease, history of diabetes type 2, history of CKD any stage, non-asthma and non-COPD chronic respiratory diseases, heart failure, liver disease, neurodevelopmental disorders, pulmonary hypertension, medical-related technological dependence), number of EUA criteria, current or former smoker during baseline, CCI, any concomitant medication (baseline period), any concomitant medication (acute period), number of healthcare encounters (baseline period), number of healthcare encounters (acute phase), vaccination status, PCR test for COVID-19 diagnosis, COVID-19 diagnosis in ER, and SARS-CoV-2 variant type based on time period of COVID-19 diagnosis

^b^Other/unknown includes "Other race", "No information", "Unknown", "Multiple races", "West Indian", "Dominican Islander", and "Hispanic”

^c^Measurements that were negative or equal to zero were removed. Measurements were then trimmed at the bottom and top one percentile (1% and 99%) to reduce the influence of outliers. If BMI value was not directly available in the patient's measurement data, it was calculated as weight/(height)2*703, where weight is reported in pounds and height is reported in inches, and where 703 is the conversion factor from metric. The weight assessment during the baseline period and closest to the index date was considered for the analysis. If multiple assessments were available on the same day, the average value was used. The maximum value for height assessed during the baseline period was considered for the analysis

^d^Assessment period included the baseline period or index date for all criteria except age, which was assessed on the index date

^e^Patients may fit into more than one category; therefore, these categories are not mutually exclusive, and the percentages do not sum to 100%

^f^Immunosuppressive disease was defined based on ICD-10, CPT-4, and/or HCPCS codes relating to any of the following: Hodgkin's lymphoma, non-Hodgkin's lymphoma, leukemia, solid cancers, HIV, autoimmune disease, solid organ transplant and/or allogenic stem cell transplant

^g^Obesity was defined based on laboratory measurements of BMI ≥ 30 or relevant ICD-10 codes

^h^Immunosuppressive treatment was defined based on HCPCS and/or NDC codes relating to systematic corticosteroid therapy or systemic non-corticosteroid immunosuppressants

^i^Medical-related technological dependence was defined based on ICD-10, CPT-4, and/or HCPCS codes relating to any of the following: respiratory aspirator, gastro- or jejunostomy, mitrofanoff, a nasogastric tube, renal replacement therapy, total parenteral nutrition, or ventricular assistance

^j^Healthcare encounters include inpatient visits (including intensive care unit visits), outpatient visits, ER visits, and other types of visits such as long-term care visits and virtual visits

^k^Assessed during the vaccination assessment period which is defined as from the start of data availability until 14 days prior to the COVID-19 diagnosis date

^l^Defined as patients who received three or more vaccinations with an mRNA vaccine (i.e., Pfizer-BioNTech [BNT162b2] or Moderna [mRNA-1273]) or two or more vaccinations with at least one of those vaccinations being with the viral vector Johnson & Johnson vaccine (JNJ-784336725)

^m^Defined as patients who received two vaccinations with an mRNA vaccine (i.e., Pfizer-BioNTech [BNT162b2] or Moderna [mRNA-1273]) or one vaccination with the viral vector Johnson & Johnson vaccine (JNJ-784336725)

^n^Defined as patients who received one vaccine with an mRNA vaccine (Pfizer-BioNTech [BNT162b2] or Moderna [mRNA-1273])

^o^Other/unknown includes patients with unknown test types and patients whose first confirmed case of COVID-19 was determined by a rapid antigen test or clinical diagnosis

*ATT* average treatment effect in the treated, *BMI* body mass index, *CCI* Charlson Comorbidity Index, *CKD* chronic kidney disease, *COPD *chronic obstructive pulmonary disease, *COVID-19* coronavirus disease 2019, *CPT-4* Current Procedural Terminology, *ER* emergency room, *EUA *emergency use authorization, *HCPCS* Healthcare Common Procedure Coding System, *HIV* human immunodeficiency virus, *ICD-10* International Classification of Diseases, Tenth Revision, *ICS* inhaled corticosteroid, *IQR* interquartile range, *mRNA *messenger ribonucleic acid, *NDC* National Drug Code, *PCR* polymerase chain reaction, *SARS-CoV-2* severe acute respiratory syndrome coronavirus 2, *SD* standard deviation, *Std. diff*. standardized difference, *US* United States

Most baseline characteristics, including COVID-19 disease history characteristics, were balanced between the treated and untreated patients after ATT weighting (i.e., standardized difference ≤ 10%). Imbalance remained for any concomitant medication during the pre-index acute phase, number of healthcare encounters during the pre-index acute phase, and baseline pregnancy. Concomitant medication and number of healthcare encounters during the pre-index acute phase were also imbalanced before weighting; however, they were not included in the PS model due to collinearity with the corresponding variable assessed during the acute phase. Instead, these variables assessed during the pre-index acute phase were included in the doubly robust Cox regression model.

### Phase 2 – comparative analysis of sotrovimab-treated versus untreated high-risk patients

#### Comparative analysis of risk of PASC

In the main initial (unadjusted) analysis, sotrovimab use was associated with an increased risk of PASC (HR 1.42 [95% CI 1.38–1.46], *p* < 0.001; Table 4). However, when doubly robust ATT weighting was applied to control for measured confounding, sotrovimab use was associated with a lower risk of PASC (HR 0.92 [0.89–0.96], *p* < 0.001). Results remained consistent (HR 0.95 [0.92–0.98, *p* < 0.001) when administrative censoring was applied to assess the impact of intercurrent events. When an alternative (more stringent) definition of PASC was applied, however, the point estimate for treatment effect was greater but this finding was not statistically significant (HR 0.89 [0.74–1.07]). The proportional hazards assumption of the Cox regression models was satisfied based on Schoenfeld residuals and graphical inspection.

**Table 4:** Comparative analysis of PASC in sotrovimab-treated high-risk versus untreated high-risk patients

	Sotrovimab-treated high-risk (*N* = 9,504)	Untreated high-risk (*N* = 619,668) *(ATT weighted sample N* = *9,523)*	*p*-value
PASC (main definition)^a^			
* N* (%)	6,217 (65.4)	324,289 (52.3)	
Unweighted HR (95% CI)	1.42 (1.38–1.46)	Reference	< 0.001*
Doubly robust ATT-weighted HR (95% CI)^b^	0.92 (0.89–0.96)	Reference	< 0.001*
PASC (administrative censoring)^c^			
* N* (%)	6,384 (67.2)	326,760 (52.7)	
Unweighted HR (95% CI)	1.46 (1.43–1.50)	Reference	< 0.001*
Doubly robust ATT-weighted HR (95% CI)^b^	0.95 (0.92–0.98)	Reference	< 0.001*
PASC (sensitivity definition)^d^			
* N* (%)	208 (2.2)	8,770 (1.4)	
Unweighted HR (95% CI)	1.53 (1.33–1.75)	Reference	< 0.001*
Doubly robust ATT-weighted HR (95% CI)^b^	0.89 (0.74–1.07)	Reference	0.21
Subgroup 1: Adult (≥ 18 years)	*N* = 9,451	*N* = 596,271 *(ATT weighted sample* *N* = *9,491)*	
PASC^a^			
* N* (%)	6,187 (65.5)	313,463 (52.6)	
Unweighted HR (95% CI)	1.41 (1.38–1.45)	Reference	< 0.001*
Doubly robust ATT-weighted HR^b^ (95% CI)	0.92 (0.89–0.95)	Reference	< 0.001*
Subgroup 2: Hospitalization and/or ICU admission during acute phase	*N* = 254	*N* = *22,710* *(ATT weighted sample* *N* = *254)*	
PASC^a^			
* N* (%)	171 (67.3)	12,162 (53.6)	
Unweighted HR (95% CI)	1.52 (1.30–1.76)	Reference	< 0.001*
Doubly robust ATT-weighted HR^b^ (95% CI)	1.14 (0.91–1.44)	Reference	0.25
Subgroup 3: Highest risk^e^	*N* = 6,846	*N* = 298,648 *(ATT weighted sample* *N* = *6,855)*	
PASC^a^			
* N* (%)	4,583 (66.9)	156,013 (52.2)	
Unweighted HR (95% CI)	1.48 (1.43–1.52)	Reference	< 0.001*
Doubly robust ATT-weighted HR^b^ (95% CI)	0.94 (0.91–0.98)	Reference	< 0.001*

^*^Indicates statistical significance (*p* < 0.05)

^a^PASC was defined as demonstration of at least one of the 26 PASC symptoms (described in [1]) at least 30 days after the first ICD-10 diagnosis code for COVID-19 (U07.1) or positive PCR or antigen test in the observation period. The observation period is defined from the end of the acute phase until the earliest of day 365 following the diagnosis date, loss to follow-up, end of N3C data availability, death, COVID-19 re-infection (defined as a second positive SARS-CoV-2 PCR/antigen test at least 45 days after the first positive SARS-CoV-2 PCR/antigen test or diagnosis of COVID-19 and after at least one negative SARS-CoV-2 PCR/antigen test), or initiation of another mAb, antiviral, or pre-exposure prophylaxis

^b^ATT-weighted Cox regression model further adjusted for covariates with a standardized difference > 10% after weighting, as well as clinically relevant covariates, which comprised any concomitant medication (acute phase before index date), number of healthcare encounters (acute phase before index date), and pregnancy

^c^PASC was defined as demonstration of at least one of the 26 PASC symptoms (described in [1]) at least 30 days after the first ICD-10 diagnosis code for COVID-19 (U07.1) or positive PCR or antigen test in the observation period. The observation period is defined from the end of the acute phase until the earliest of day 365 following the diagnosis date, loss to follow-up, end of N3C data availability, or death

^d^PASC was defined based on: (1) the use of ICD-10 diagnosis code U09.9 on or after October 1, 2021 or the use of ICD-10 diagnosis code B94.8 prior to October 1, 2021 at least 30 days after the first ICD-10 diagnosis code for COVID-19 (U07.1) or positive PCR or antigen test; and/or (2) at least one visit to a PASC specialty clinic 30 days after the first ICD-10 diagnosis code for COVID-19 (U07.1) or positive PCR or antigen test. The observation period is defined from the end of the acute phase until the earliest of day 365 following the diagnosis date, loss to follow-up, end of N3C data availability, death, COVID-19 re-infection (defined as a second positive SARS-CoV-2 PCR/antigen test at least 45 days after the first positive SARS-CoV-2 PCR/antigen test or diagnosis of COVID-19 and after at least one negative SARS-CoV-2 PCR/antigen test), or initiation of another mAb, antiviral, or pre-exposure prophylaxis

^e^The highest risk subgroup included patients with the following: immunocompromised/immunosuppressant treatment, age ≥ 65 years, end-stage renal disease, transplant, or late-stage cancer

*ATT* average treatment effect in the treated, *CI* confidence interval, *COVID-19* coronavirus disease 2019, *HR* hazard ratio, *ICD-10* International Classification of Diseases, Tenth Revision, *ICU* intensive care unit, *mAb* monoclonal antibody, *N3C* National COVID Cohort Collaborative, *PASC* post-acute sequelae of COVID-19, *PCR* polymerase chain reaction, *SARS-CoV-2* severe acute respiratory syndrome coronavirus 2

In the multivariable Cox regression model approach, sotrovimab use was independently associated with a significantly decreased risk of PASC (adjusted HR 0.96 [0.94–0.99], *p* < 0.001; Table 5), demonstrating that the main finding was independent of the method of confounding control. Demographic characteristics that were associated with higher risk of PASC included age 18–64 years, female sex, White race and non-Hispanic or Latino ethnicity, and residence in the Midwest, South, and other/unknown regions (compared with the West). Clinical characteristics associated with higher risk of PASC were being obese or overweight, comorbidities (either alone [a history of type 2 diabetes, stage ≥ 3 chronic kidney disease, acute or acute-on-chronic respiratory failure, heart failure, pulmonary hypertension, having > 1 EUA criteria] or summarised by a high CCI), being a current or former smoker, use of concomitant medication during the baseline period and/or acute phase, healthcare utilization (HCRU) during the baseline period and/or acute phase, COVID-19 diagnosis by PCR test, and COVID-19 diagnosis in the ER setting.

**Table 5:** Comparative analysis of PASC^a^ in sotrovimab-treated high-risk versus untreated high-risk patients using multivariable Cox regression model^b,c,d^

	HR (95% CI)^e^	*p*-value
Unadjusted		
Treated versus untreated (reference)	1.42 (1.38–1.46)	< 0.001*
Adjusted		
Treated versus untreated (reference)	0.96 (0.94–0.99)	< 0.001*
Age at index date		
< 18 years	0.98 (0.96–0.996)	0.02*
18–64 years	Reference	
≥ 65 years	0.83 (0.82–0.83)	< 0.001*
Sex		
Female	Reference	
Male	0.85 (0.84–0.86)	< 0.001*
Other/unknown	0.95 (0.69–1.30)	0.73
Race		
White	Reference	
Black or African American	0.99 (0.98–1.00)	0.24
Asian or Pacific Islander	0.90 (0.88–0.92)	< 0.001*
Other/unknown^f^	0.99 (0.97–1.00)	0.006
Ethnicity		
Non-Hispanic or Latino	Reference	
Hispanic or Latino	0.93 (0.92–0.94)	< 0.001*
Other/unknown	0.98 (0.97–0.99)	< 0.001*
US geographic region		
West	Reference	
Midwest	1.03 (1.01–1.04)	< 0.001*
South	1.15 (1.13–1.16)	< 0.001*
Northeast	0.87 (0.86–0.89)	< 0.001*
Other/unknown	1.11 (1.10–1.13)	< 0.001*
BMI^g^		
< 18.5 (underweight)	0.98 (0.93–1.03)	0.40
18.5–24.9 (normal)	Reference	
25.0–29.9 (overweight)	1.04 (1.02–1.05)	< 0.001*
≥ 30 (obese)	1.05 (1.04–1.07)	< 0.001*
Unknown	0.96 (0.95–0.98)	< 0.001*
EUA criteria^h,i^		
Cardiovascular disease (including congenital heart disease) or hypertension	0.99 (0.98–0.997)	0.02*
Immunosuppressive disease^j^	0.89 (0.88–0.89)	< 0.001*
History of diabetes type 2	1.07 (1.06–1.08)	< 0.001*
History of CKD (stage ≥ 3)	1.00 (0.99–1.02)	0.99
Acute or acute-on-chronic respiratory failure	1.05 (1.03–1.06)	< 0.001*
Heart failure	1.03 (1.01–1.05)	< 0.001*
Liver disease	0.95 (0.94–0.97)	< 0.001*
Pregnancy	0.70 (0.69–0.71)	< 0.001*
Neurodevelopmental disorders	0.97 (0.96–0.99)	< 0.001*
Pulmonary hypertension	1.05 (1.02–1.08)	< 0.001*
A medical-related technological dependence^k^	0.83 (0.80–0.86)	< 0.001*
Number of EUA criteria		
1, *n* (%)	Reference	
2, *n* (%)	1.32 (1.31–1.33)	< 0.001*
3, *n* (%)	1.57 (1.55–1.59)	< 0.001*
4, *n* (%)	1.82 (1.79–1.85)	< 0.001*
≥ 5, *n* (%)	2.12 (2.07–2.16)	< 0.001*
Smoking status during baseline		
Non-smoker	Reference	
Current or former smoker	1.07 (1.06–1.08)	< 0.001*
CCI	1.01 (1.01–1.02)	< 0.001*
Concomitant medication		
Baseline period		
Did not receive any medication	Reference	
Received any medication	1.09 (1.07–1.10)	< 0.001*
Acute phase		
Did not receive any medication	Reference	
Received any medication	1.17 (1.16–1.18)	< 0.001*
Healthcare utilization^l^		
Number of healthcare encounters during baseline^m^	1.00 (1.00–1.00)	< 0.001*
Number of healthcare encounters during acute phase^m^	1.03 (1.03–1.03)	< 0.001*
Type of test used to diagnose COVID-19		
Other / unknown test	Reference	
PCR test	1.03 (1.02–1.04)	< 0.001*
SARS-CoV-2 variant type based on time period of COVID-19 diagnosis^n^		
Delta	0.93 (0.92–0.94)	< 0.001*
Omicron BA.1 (before Paxlovid® availability)^o^	0.96 (0.95–0.97)	< 0.001*
Omicron BA.1 (after Paxlovid® availability)^o^	Reference	
Omicron BA.2	1.01 (0.99–1.03)	0.46
Setting of COVID-19 diagnosis		
Diagnosis of COVID-19 in other settings	Reference	
Diagnosis of COVID-19 in the emergency room	1.07 (1.06–1.08)	< 0.001*

^*^Indicates statistical significance (*p* < 0.05)

^a^PASC was defined as demonstration of at least one of the 26 PASC symptoms (described in [1]) at least 30 days after the first ICD-10 diagnosis code for COVID-19 (U07.1) or positive PCR or antigen test in the observation period. The observation period was defined from the end of the acute phase until the earliest of day 365 following the diagnosis date, loss to follow-up, end of N3C data availability, death, COVID-19 re-infection (i.e., second positive SARS-CoV-2 PCR/antigen test at least 45 days after the first positive SARS-CoV-2 PCR/antigen test or diagnosis of COVID-19 and after at least one negative SARS-CoV-2 PCR/antigen test), or initiation of another mAb, antiviral, or pre-exposure prophylaxis

^b^The multivariable Cox regression model adjusted for covariates included in the propensity score used to generate ATT weights and the Cox regression model from the main analysis, which included age at index date, sex, race, ethnicity, US geographic region, BMI, EUA criteria (cardiovascular disease or hypertension, immunosuppressive disease, history of diabetes type 2, history of CKD any stage, non-asthma and non-COPD chronic respiratory diseases, heart failure, liver disease, neurodevelopmental disorders, pulmonary hypertension, medical-related technological dependence), number of EUA criteria, current or former smoker during baseline, CCI, any concomitant medication (baseline period), any concomitant medication (acute period), number of healthcare encounters (baseline period), number of healthcare encounters (acute phase), PCR test for COVID-19 diagnosis, COVID-19 diagnosis in the emergency room, SARS-CoV-2 variant type based on time period of COVID-19 diagnosis, and pregnancy

^c^Out of 9,504 treated patients, 6,217 (65.4%) had PASC in the observation period. Out of 619,668 untreated patients, 324,289 (52.3%) had PASC in the observation period

^d^E-values in our models for risk of PASC ranged from 1.11 to 4.42, which were relatively large in magnitude compared with the magnitude of association for other adjusted covariates, suggesting that it was unlikely that unmeasured confounding would have a substantially greater effect on the outcomes than these adjusted covariates

^e^HR (95% CI) values that were rounded to “1.00” were presented with 3 decimal places to avoid confusion

^f^Other/unknown includes "Other race", "No information", "Unknown", "Multiple races", "West Indian", "Dominican Islander", and "Hispanic"

^g^Measurements that were negative or equal to zero were removed. Measurements were then trimmed at the bottom and top one percentile (1% and 99%) to reduce the influence of outliers. If BMI value was not directly available in the patient's measurement data, it was calculated as weight/(height)2*703, where weight is reported in pounds and height is reported in inches, and where 703 is the conversion factor from metric. The weight assessment during the baseline period and closest to the index date was considered for the analysis. If multiple assessments were available on the same day, the average value was used. The maximum value for height assessed during the baseline period was considered for the analysis

^h^Assessment period included the baseline period or index date for all criteria except age, which was assessed on the index date

^i^Patients may fit into more than one category; therefore, these categories are not mutually exclusive, and the percentages do not sum to 100%

^j^Immunosuppressive disease was defined based on ICD-10, CPT-4, and/or HCPCS codes relating to any of the following: Hodgkin's lymphoma, non-Hodgkin's lymphoma, leukemia, solid cancers, HIV, autoimmune disease, solid organ transplant and/or allogenic stem cell transplant

^k^Medical-related technological dependence was defined based on ICD-10, CPT-4, and/or HCPCS codes relating to any of the following: respiratory aspirator, gastro- or jejunostomy, mitrofanoff, a nasogastric tube, renal replacement therapy, total parenteral nutrition, or ventricular assistance

^l^For patients with more than two visits on the same day (regardless of types of encounters), only one encounter was included

^m^Healthcare encounters include inpatient visits (including ICU visits), outpatient visits, emergency room visits, and other types of visits such as long-term care visits and virtual visits

^n^The SARS-CoV-2 variant types were defined by the time of the patient's confirmed initial COVID-19 diagnosis period

^o^Omicron BA.1 time period was separated into two time periods before and after Paxlovid® availability on December 21, 2021

*ATT* average treatment effect in the treated, *BMI* body mass index, *CCI* Charlson Comorbidity Index, *CI* confidence interval, *CKD* chronic kidney disease, *COVID-19* coronavirus disease 2019, *CPT-4* Current Procedural Terminology, *EUA* emergency use authorization, *HCPCS *Healthcare Common Procedure Coding System, *HIV* human immunodeficiency virus, *HR *hazard ratio, *ICD-10* International Classification of Diseases, Tenth Revision, *ICU* intensive care unit, *mAb* monoclonal antibody, *N3C *National COVID Cohort Collaborative, *PASC* post-acute sequelae of COVID-19, *PCR *polymerase chain reaction, *SARS-CoV-2* severe acute respiratory syndrome coronavirus 2, *US* United States

#### Subgroups of interest (exploratory objective)

Doubly robust ATT-weighted Cox regression findings in the adult (HR 0.92 [0.89–0.95], *p* < 0.001) and highest-risk (HR 0.94 [0.91–0.98], *p* < 0.001) subgroups were consistent with the main analysis, with a significantly reduced risk of PASC among sotrovimab-treated patients (Table 4). No significant difference between the cohorts was observed with respect to the risk of PASC among the subgroup of patients with hospitalization and/or ICU admission during the acute phase.

### Phase 2 – untreated high-risk versus untreated non-high-risk patients

Results for the comparisons of the 1,715,283 untreated high-risk patients with the 1,813,311 untreated non-high-risk patients with COVID-19 are reported in the Supplementary Appendix.

## Discussion

The prevalence of PASC remains high, affecting an estimated 10–30% of the global population [6]. Research has shown that PASC increases HCRU and costs [5], reinforcing the need for improved prevention. Effective treatments to prevent the long-term sequelae of COVID-19 could therefore provide value to patients, clinicians, and public health programs.

To our knowledge, this was the first study to evaluate the impact of sotrovimab on the risk of PASC, defined as a diagnosis of ≥ 1 of 26 PASC symptoms ≥ 30 days after the first COVID-19 diagnosis. The results demonstrated that, among high-risk COVID-19 patients, sotrovimab use was associated with an 8% decreased risk of developing PASC compared with untreated patients. Furthermore, the effect of sotrovimab treatment among high-risk COVID-19 patients remained largely consistent across different PASC definitions, follow-up durations, and analytical approaches, supporting the robustness of the findings. During the study period, sotrovimab may therefore have been an effective option for PASC prevention in a high-risk population.

Others have studied the real-world effectiveness of COVID-19 vaccines and mAbs (products not specified) against PASC using data from an observational registry for a diverse US metropolitan population (*n* = 53,239) [19]. Both vaccinated breakthrough cases (vs unvaccinated) and mAb-treated patients (vs untreated) had lower likelihoods for developing PASC. For all symptoms, vaccination was associated with a lower likelihood of experiencing PASC compared with mAb treatment [19]. PASC has been postulated to have numerous underlying pathologies, including a heightened and prolonged immune response, and autoantibody-driven immunomodulatory process, and active or persistent SARS-CoV-2 infection [20–22]. Based on these potential pathologies, it is plausible that boosted immunity may explain the prevention of PASC through vaccination or mAbs.

Several studies have assessed the impact of antiviral agents on the development of PASC. A single-center, prospective cohort study (*n* = 760) reported that nirmatrelvir/ritonavir reduced the risk of PASC in patients six months after hospital discharge following admission with COVID-19 [23]. Another study, using data from non-hospitalized US veterans, showed no differences between participants treated with nirmatrelvir/ritonavir (*n* = 9,593) and their matched untreated comparators in the incidence of post-COVID conditions (PCC), except for lower combined risk for venous thromboembolism and pulmonary embolism [24]. Xie et al. reported that treatment with nirmatrelvir within five days of a positive SARS-CoV-2 test result was associated with reduced risk of PCC (defined as a weighted sum of 13 PASC symptoms) across the risk spectrum and regardless of vaccination status and history of prior infection [25]. Another study of non-hospitalized patients with COVID-19 found that nirmatrelvir treatment during acute infection was not associated with patient self-reported PASC symptoms more than 90 days post-infection [26].

There is currently no widely accepted definition of PASC, and hence the choice of endpoint/definition used has the potential to impact our findings. The literature review conducted in Phase 1 of our study showed considerable heterogeneity in definitions of PASC. The presence of at least one of 26 PASC symptoms outlined in Bull-Otterson et al. [1] served as the basis for the main PASC definition used in this study. A study from the RECOVER Initiative constructed a PASC score as a weighted sum of 12 symptoms and identified an optimal score threshold, above which patients were classified as having PASC [27]. A systematic review and meta-analysis of 41 studies defined PASC as ≥ 1 new or persisting symptom of PASC at least 28 days after the first test/diagnosis, date of hospitalization, discharge date, or date of clinical recovery/negative test [28], while another meta-analysis defined PASC as one or more symptoms ongoing for at least 28 days [29]. Although these meta-analyses used a broader definition of PASC compared with our study, their CIs are consistent with the percentage of patients with PASC in our study population. In another N3C-based study, PASC was defined as the presence of a long-COVID diagnosis code or visits to a long-COVID clinic [30], which was consistent with the sensitivity definition of PASC in this study. Moreover, the study identified 0.8% of patients with PASC from the N3C population using this definition, which was also consistent with that reported in our study (0.8% – definition 2). Pfaff et al. aimed to characterize the N3C population based on a U09.9 post-COVID-19 diagnosis code [31], which was also included in our sensitivity definition. The code was shown, however, to be disproportionately used among patients who were female, White and non-Hispanic, suggesting disparities in practice patterns related to diagnosis of PASC [31]. The main definition of PASC used in our study was selected systematically to leverage consistency across published definitions and to capture a broad range of PASC symptoms based on the available data, and the inclusion of an alternative definition as a sensitivity analysis allowed us to explore the effect of a more stringent PASC definition on the study findings. In effect, however, these may represent sensitivity over specificity: the broad definition clearly captured more PASC patients, but the potential for false positives would increase, biasing the findings toward the null (assuming non-differential misclassification from unrelated diagnostic “noise”), whereas the stringent definition likely only captured a small number of “true” PASC patients, leading to a largely consistent effect estimate but a lack of precision. Future studies may want to consider a “happy medium” definition which sits between broad and narrow.

Results from subgroup analyses, which may have provided additional context on the role of sotrovimab, were largely driven by sample size. In the adult subgroup, comparative results were similar to those from the overall sample and showed a protective effect of sotrovimab on PASC risk. This is unsurprising, however, given that adults comprised 99.4% of sotrovimab-treated patients and 96.2% of untreated patients in this study. Similarly, 72.0% of sotrovimab-treated patients and 48.2% of untreated patients were included in the highest risk subgroup analysis by virtue of having at least one EUA condition; therefore, the effect of sotrovimab against PASC in this sub-population may also be expected. The subgroup of patients with hospitalization and/or ICU admission during the acute phase represented only 2.7% and 3.7% of all treated and untreated patients, respectively, and this might have explained the observed lack of effect in this analysis. Alternatively, the unadjusted effect estimate was in line with those observed for the overall study and the other subgroups but, unlike elsewhere, the effect estimate remained above one when doubly robust ATT-weighting was applied; this suggests that there may have been channeling bias for this sicker subset that was not accounted for in the analysis.

Results from the sensitivity analysis using the multivariable Cox regression approach were similar and significant, further supporting the robustness of findings. These results were expected, as high-risk patients had a larger number of health conditions and likely had more severe COVID-19 disease than the non-high-risk cohort. This is consistent with previous studies, including a US study that reported an increased prevalence of PASC among high-risk patients [3]. When a more stringent definition of PASC was applied, the effect estimate was greater although was not statistically significant; this may be explained by the smaller number of events for sotrovimab-treated and untreated patients in this analysis (2.2% and 1.4%, respectively) versus the main analysis (65.4% and 52.3%, respectively).

The current study also included a supplementary analysis of PASC risk in untreated high-risk compared with untreated non-high-risk patients. While control for all confounders is impossible in such an analysis, the elevated risk of PASC among high-risk compared with non-high-risk patients further highlights the importance of effective treatment options for PASC prevention in high-risk populations, among whom the burden of PASC may be substantial.

### Study limitations

Several additional limitations of this study warrant consideration. The study focused on patients with confirmed COVID-19 and hence the findings may not be generalizable to patients with unconfirmed disease, who may differ from those with confirmed COVID-19 in terms of severity and/or time since disease onset. Similarly, findings may not be generalisable beyond the US.

As this is an observational study that relies on electronic health record data, caution should be exercised when interpreting results due to the potential for incomplete and inaccurate data as well as bias from residual or unmeasured confounding. Such bias, if present, is likely to be non-differential with regards to treatment cohort. Channeling bias may be present, particularly for the high-risk population, and is a potential source of residual confounding; higher-risk patients may be channelled to receive sotrovimab, which would tend to attenuate the protective effect of sotrovimab on PASC risk. Immortal time bias may also have been present as the study required patients to survive and have continuous follow-up through the acute phase; the impact of immortal time bias is expected to be minimal and non-differential. Also, the algorithm for identification of patients with COVID-19 included the criterion of two weak positive diagnosis codes (based on codes related to symptoms of COVID-19) during the same encounter or on the same day. This was included in order to capture COVID-19 cases that occurred before the presence of a diagnosis code. While we recognize the potential for this to introduce patients with non-COVID infections, it would likely bias results to the null since treatment was based on clinical need and not test positivity.

The present study was conducted at a time when early variants of Omicron (BA.1 and BA.2) were emerging in the US, for which reduced in vitro neutralization of sotrovimab has been demonstrated [13]. However, it is unclear how well in vitro data reflect the real-world clinical effectiveness of sotrovimab [32]; sotrovimab has the ability to mediate potent FC-effector functions, such as antibody-dependent cellular cytotoxicity and antibody-dependent cellular phagocytosis to target the virus, but this is not measured in standard neutralization assays [33–36]. Indeed, several other studies conducted in the US and elsewhere have demonstrated the real-world clinical benefit of sotrovimab during periods of Omicron subvariant predominance [37–41].

Cohort definitions were based on assumptions that patients who received sotrovimab have mild/moderate COVID-19 per the sotrovimab label, while patients in the untreated cohort (for comparison with the treated cohort) were assumed to have mild/moderate COVID-19 as both cohorts were required to not be hospitalized between the date of COVID-19 diagnosis and the index date. These assumptions may not accurately capture mild/moderate disease, the definition of which is difficult to assess without complete data on symptoms experienced.

Patients who were previously administered a mAb, antiviral medicine or pre-exposure prophylaxis during the baseline period were excluded from our analyses to ensure that sotrovimab was the only treatment exposure. We were therefore unable to assess potential for synergistic or additive effects, and study findings may not be generalizable to patients who have received multiple therapeutics.

Finally, and as acknowledged above, PASC is a complex syndrome with a large degree of heterogeneity in its definition. The definition chosen for our study may therefore have increased the potential for misclassification of the study outcomes. However, the results of the sensitivity analysis that employed a more stringent definition were generally consistent with those from the main analysis.

## Conclusions

This retrospective observational study found that in patients at high risk for severe COVID-19, treatment with sotrovimab within 10 days of COVID-19 diagnosis was associated with lower risk of PASC. These findings suggest that the benefits of early treatment with sotrovimab may extend beyond the acute phase of COVID-19 and contribute to the prevention of PASC symptoms in a population with substantial disease burden.

## Supplementary Information

Below is the link to the electronic supplementary material.

Supplementary file1 (DOCX 228 KB)

## Data Availability

For requests for access to anonymized subject level data, please contact the Corresponding Author.
