# Supplementary material for: Impact of treatment of COVID-19 with sotrovimab on post-acute sequelae of COVID-19 (PASC): an analysis of National COVID Cohort Collaborative (N3C) data
Source: Infection. 2025 Mar 22;53(5):1833–49. doi: 10.1007/s15010-025-02505-z (PMC12460566; doi:10.1007/s15010-025-02505-z)
Supplement: Supplementary file 1 — Supplementary file1 (DOCX 228 KB) [file 15010_2025_2505_MOESM1_ESM.docx]

# Supplementary Appendix

**Impact of treatment of COVID-19 with sotrovimab on post-acute sequelae of COVID-19 (PASC): an analysis of National COVID Cohort Collaborative (N3C) data**

Myriam Drysdale^1^, Rose Chang^2^, Tracy Guo^2^, Mei Sheng Duh^2^, Jennifer Han^3^, Helen Birch^4^, Catherine Sharpe^1^, Daisy Liu^2^, Sarah Kalia^2^, Melissa Van Dyke^4^, Maral DerSarkissian^5^, Iain A. Gillespie^4^

^1^GSK, Brentford, Middlesex, UK

^2^Analysis Group, Inc., Boston, MA, USA

^3^GSK, Collegeville, PA, USA

^4^GSK, Stevenage, Hertfordshire, UK

^5^Analysis Group, Inc., Los Angeles, CA, USA

**ORCID IDs**

Myriam Drysdale: 0000-0002-8994-2816

Jennifer Han: 0000-0003-4744-8067

Helen J. Birch: 0000-0002-4924-4810

Iain A. Gillespie: 0000-0002-2265-9506

**Corresponding author:**

Myriam Drysdale, PhD

Director, Epidemiology

GSK

980 Great West Road, Brentford, TW8 9GS, UK

myriam.g.drysdale@gsk.com

## Phase 1 – literature search

## Potential definition(s) of PASC were identified through a brief review of 13 observational studies [1-13], seven systematic reviews and/or meta-analysis [14], and two official guidelines on definitions of PASC [15, 16].

## Overall, there was substantial heterogeneity in published definitions of PASC, including the characterization of PASC and duration of the acute phase. Several definitions of PASC utilized by the RECOVER Initiative, a National Institutes of Health-funded initiative that aims to understand, treat, and prevent PASC through a wide variety of research, were identified. The definitions for PASC used by the RECOVER Initiative are outlined in the first four definitions below. In addition, an alternative definition that differs with respect to the duration of symptoms following coronavirus disease 2019 (COVID-19) diagnosis was assessed. Specifically, it represents the midway point (i.e., 60 days) between the Centers for Disease Control and Prevention (CDC)-endorsed 30-day definition and the World Health Organization (WHO)-recommended 90-day definition, which are consistent with definitions 3 and 4 from RECOVER below, respectively. Therefore, the following five definitions of PASC were evaluated:

## 1. Use of International Classification of Diseases, Tenth Revision, Clinical Modification (ICD-10-CM) diagnosis code U09.9 at least 30 days after the first ICD-10 diagnosis code for COVID-19 (U07.1) or a positive polymerase chain reaction (PCR) or antigen test for COVID-19 [11]: The ICD-10-CM diagnosis code U09.9 (i.e., post COVID-19 condition, unspecified) was not available until October 1, 2021. To capture patients with PASC that occurred before this time period, ICD-10-CM diagnosis code B94.8 (i.e., sequelae of other specified infectious and parasitic disease) amongst patients with COVID-19 (i.e., patients with ICD-10-CM COVID-19 diagnosis code U07.1 from an inpatient or emergency visit, or a positive severe acute respiratory syndrome coronavirus 2 [SARS-CoV-2] PCR or antigen test) was used based on the CDC’s recommendation.

## 2. Referral to a long-COVID clinic: Patients with PASC were identified based on visits to a long-COVID specialty clinic at least once 30 days after diagnosis of COVID-19.

## 3. Demonstration of PASC at least 30 days after the first ICD-10 diagnosis code for COVID-19 (U07.1) or a positive PCR or antigen test for COVID-19: Bull-Otterson et al. followed patients from day 30 to day 365 after initial COVID-19 diagnosis to assess the incidence of 26 clinical conditions that have been often attributed to PASC [2]. This 30-day duration of the acute phase of COVID-19 is endorsed by the CDC and is utilized by various other studies [1, 2, 7-9, 11, 17-20]. PASC was defined as the occurrence of at least one of the 26 symptoms at least 30 days after COVID-19 diagnosis, as described by Bull-Otterson et al [2].

## 4. Demonstration of PASC at least 60 days after the first ICD-10 diagnosis code for COVID-19 (U07.1) or a positive PCR or antigen test for COVID-19 [19, 21-23]: PASC was defined as the occurrence of at least one of the 26 symptoms at least 60 days after COVID-19 diagnosis, as described by Bull-Otterson et al [2].

## 5. Demonstration of PASC at least 90 days after the first ICD-10 diagnosis code for COVID-19 (U07.1) or a positive PCR or antigen test for COVID-19: Pfaff and colleagues used machine learning techniques to identify characteristics of patients with PASC and those likely to have PASC using the National COVID Cohort Collaborative (N3C) data, defining potential PASC as symptoms that occurred in non-deceased adult patients with either an ICD-10-CM COVID-19 diagnosis code (U07.1) from an inpatient or emergency visit, or a positive SARS-CoV-2 PCR or antigen test, and for whom at least 90 days have passed since the COVID-19 diagnosis date [10]. This 90-day duration of the acute phase of COVID-19 is endorsed by the WHO and is utilized in various other studies examining PASC [4-6, 12, 24, 25]. PASC was defined as the occurrence of at least one of the 26 symptoms at least 90 days after COVID-19 diagnosis, as described by Bull-Otterson et al [2].

## Supplementary Table S1 Inclusion and exclusion criteria

| **Inclusion** | **Exclusion** |
| --- | --- |
| - Age ≥12 years on the index date - Confirmed COVID-19 by PCR or antigen test or presence of diagnosis code U07.1 (COVID-19, virus identified) - Sufficient data to allow at least 12 months of baseline period prior to the index date - Survival and continuous follow-up through the acute phase - High risk for progression to severe COVID-19 (fulfilling at least one of the EUA criteria for sotrovimab^a^) - Sotrovimab-treated patients only: treated with sotrovimab in an outpatient or ER setting within 10 days of COVID-19 diagnosis | - Previously administered mAb, antiviral, or tixagevimab/cilgavimab during the baseline period - Re-infection with COVID-19^b^ - Not hospitalized between the date of COVID-19 diagnosis and the index date - Sotrovimab-treated patients only: additional sotrovimab administration ≥2 days after the index date during the acute phase |

*Abbreviations* CKD, chronic kidney disease; COPD, chronic obstructive pulmonary disease; COVID-19, coronavirus disease 2019; ER, emergency room; EUA, emergency use authorization; ICS, inhaled corticosteroid; mAB, monoclonal antibody; PCR, polymerase chain reaction; SARS-CoV-2, severe acute respiratory syndrome coronavirus 2

^a^Includes age ≥65 years, obesity, pregnancy, history of CKD, history of diabetes type 1 or type 2, immunosuppressive disease/treatment, cardiovascular disease (including congenital heart disease) or hypertension, chronic lung disease (COPD or asthma), sickle cell disease, neurodevelopmental disorders, medical-related technological dependence, liver disease, anti-diabetic therapies, acute or acute-on-chronic respiratory failure, pulmonary hypertension, heart failure, acquired heart disease, non-asthma/COPD chronic respiratory diseases, ICS-containing therapies

^b^Second positive SARS-CoV-2 PCR/antigen test at least 45 days after COVID-19 diagnosis and after at least one negative SARS-CoV-2 PCR/antigen test

## Phase 2 – untreated high-risk versus untreated non-high-risk patients

Analyses were conducted among a random sample of 25% of the eligible population (*n*= 446,425 patients for untreated high-risk and *n* = 433,756 patients for untreated non-high-risk).

***Baseline characteristics***

Baseline characteristics are shown in **Supplementary Table S3**. High-risk untreated patients were older than non-high-risk untreated patients (mean 46 vs 36 years, respectively). Nearly all demographic and COVID-19 disease history characteristics were balanced between the two cohorts after average treatment effect in the treated (ATT) weighting (standardized difference ≤10%). To maintain the distinction between the high-risk versus non-high-risk cohorts, clinical characteristics (emergency use authorization criteria, smoking history, Charlson comorbidity index, concomitant medication) and healthcare utilization variables were not included in the propensity scores model for ATT weighting, despite being imbalanced between the groups.

***Comparative analysis of risk of PASC***

In the main initial (unadjusted) analysis, untreated high-risk untreated patients had an increased risk of PASC (hazard ratio [HR] 2.49 [95% confidence interval 2.47–2.51], *p*< 0.001). The doubly robust ATT-weighted HR was 2.08 (2.06–2.10) (*p* < 0.001) (**Supplementary Table S4**), indicating a higher risk of PASC among untreated high-risk patients than untreated non-high-risk patients. Results remained consistent in the sensitivity analysis using PASC definition 1 and/or 2 (**Supplementary Table S4**). In the multivariable Cox regression model, the adjusted HR was 2.23 (2.21–2.24) (*p* < 0.001), which was similar to the main analysis (**Supplementary Table S5**). Characteristics associated with higher risk of PASC included age ≥18 years (compared with children age <18 years), female sex (compared to males), White race and non-Hispanic or Latino ethnicity (compared with other races and ethnicities), residence in the West (compared with residence in the Midwest and Northeast), being obese or overweight (compared with being normal weight), having the Omicron BA.2 variant (compared with Omicron BA.1 infection after Paxlovid^®^ availability), being a current or former smoker, and setting of COVID-19 diagnosis in the ER.

## Supplementary Table S2 Overlap between definitions of PASC in the 90-day acute-phase sample^a^

|  | **Number with acute phase corresponding to definition** | **Number with 90-day acute phase** | **Number with PASC during the observation phase, *n* (% with 90-day acute phase)^b^** | **Pairwise overlap in patients meeting each definition, n (% of total)^c^** | | | | |
| --- | --- | --- | --- | --- | --- | --- | --- | --- |
|  |  |  |  | 90–100%  80–90%  40–80%  <40% | | | | |
|  |  |  |  | **Definition 1^d^** | **Definition 2^e^** | **Definition 3^f^** | **Definition 4^g^** | **Definition 5^h^** |
| **Untreated high-risk patients (for comparison with untreated non-high-risk patients: COVID-19 diagnosis on or after March 1, 2020)** | | | | | | | | |
| Definition 1^d^ | 1,715,283 | 1,616,682 | 15,657 (1.0) | – | 1,147 (7.3) | 13,991 (89.4) | 12,868 (82.2) | 11,844 (75.6) |
| Definition 2^e^ | 1,715,283 | 1,616,682 | 2,305 (0.1) | 1,147 (49.8) | – | 2,055 (89.2) | 1,920 (83.3) | 1,779 (77.2) |
| Definition 3^f^ | 1,715,283 | 1,616,682 | 823,335 (50.9) | 13,991 (1.7) | 2,055 (89.2) | – | 755,889 (91.8) | 689,914 (83.8) |
| Definition 4^g^ | 1,663,918 | 1,616,682 | 755,885 (46.8) | 12,868 (1.7) | 1,920 (0.3) | 755,889 (100.0) | – | 689,914 (91.3) |
| Definition 5^h^ | 1,616,682 | 1,616,682 | 689,914 (42.7) | 11,844 (1.7) | 1,779 (0.3) | 689,914 (100.0) | 689,914 (100.0) | – |
| **Untreated non-high-risk patients** | | | | | | | | |
| Definition 1^d^ | 1,813,311 | 1,731,467 | 6,564 (0.4) | – | 570 (8.7) | 5,547 (84.5) | 4,918 (74.9) | 4,445 (67.7) |
| Definition 2^e^ | 1,813,311 | 1,731,467 | 1,013 (0.1) | 570 (56.3) | – | 842 (83.1) | 757 (74.7) | 688 (67.9) |
| Definition 3^f^ | 1,813,311 | 1,731,467 | 464,836 (26.8) | 5,547 (1.2) | 842 (0.2) | – | 427,550 (92.0) | 390,321 (84.0) |
| Definition 4^g^ | 1,773,706 | 1,731,467 | 427,550 (24.7) | 4,918 (1.2) | 757 (0.2) | 427,550 (100.0) | – | 390,321 (91.3) |
| Definition 5^h^ | 1,731,467 | 1,731,467 | 390,321 (22.5) | 4,445 (1.1) | 688 (0.2) | 390,321 (100.0) | 390,321 (100.0) | – |

*Abbreviations* COVID-19, coronavirus disease 2019; ICD-10, International Classification of Diseases, Tenth Revision; mAb, monoclonal antibody; N3C, National COVID Cohort Collaborative; PASC, post-acute sequelae of COVID-19; PCR, polymerase chain reaction; SARS-CoV-2, severe acute respiratory syndrome coronavirus 2

^a^Cohort sample size depends on acute-phase length, which varies across PASC definitions (i.e., 30, 60, and 90 days); however, the same population should be used to assess overlap between definitions such that the percentages could be directly compared with each other. Since patients included in the 30-day and 60-day acute-phase samples would also be included in the 90-day acute-phase sample, the 90-day acute-phase sample was used for this analysis

^b^The observation period was defined from the end of the acute phase until the earliest of day 365 following the diagnosis date, loss to follow-up, end of N3C data availability, death, COVID-19 re-infection (second positive SARS-CoV-2 PCR/antigen test at least 45 days after the first positive SARS-CoV-2 PCR/antigen test or diagnosis of COVID-19, and after ≥1 negative SARS-CoV-2 PCR/antigen test), or initiation of another mAb, antiviral, or pre-exposure prophylaxis.

^c^The pairwise overlap percentage is based on row total (i.e., the number of patients meeting the PASC definition in each column divided by the total number of patients meeting the PASC definition in each row)

^d^Definition 1 was defined as the use of U09.9 on or after October 1, 2021, or the use of B94.8 prior to October 1, 2021 at least 30 days after the first ICD-10 diagnosis code for COVID-19 (U07.1) or positive PCR or antigen test in the observation period

^e^Definition 2 was defined as at least one visit to a long-COVID specialty clinic 30 days after the first ICD-10 diagnosis code for COVID-19 (U07.1) or positive PCR or antigen test in the observation period

^f^Definition 3 was defined as demonstration of at least one of the 26 PASC symptoms (described in [1]) at least 30 days after the first ICD-10 diagnosis code for COVID-19 (U07.1) or positive PCR or antigen test in the observation period

^g^Definition 4 was defined as demonstration of at least one of the 26 PASC symptoms (described in [1]) at least 60 days after the first ICD-10 diagnosis code for COVID-19 (U07.1) or positive PCR or antigen test in the observation period

^h^Definition 5 was defined as demonstration of at least one of the 26 PASC symptoms (described in [1]) at least 90 days after the first ICD-10 diagnosis code for COVID-19 (U07.1) or positive PCR or antigen test in the observation period

## Supplementary Table S3 Baseline characteristics of untreated high-risk versus untreated non-high-risk patients prior to and after ATT weighting

|  | **Unweighted sample** | | | **Weighted sample^b^** | | |
| --- | --- | --- | --- | --- | --- | --- |
|  | **Untreated high-risk**  **(*N* = 446,425)** | **Untreated non-high-risk**  **(*N* = 433,756)^a^** | **Std. diff. (%)** | **Untreated high-risk**  **(*N* = 446,425)** | **Untreated non-high-risk**  **(*N* = 450,399)^a^** | **Std. diff. (%)** |
| **Demographic characteristics** |  |  |  |  |  |  |
| Age at index date (years) |  |  |  |  |  |  |
| <65, *n* (%) | 270,077 (60.5) | 433,756 (100.0) | – | 270,077 (60.5) | 450,399 (100.0) | – |
| 12–17, *n* (%) | 11,861 (2.7) | 47,552 (11.0) | – | 11,861 (2.7) | 17,305 (3.8) | – |
| 18–64, *n* (%) | 258,216 (57.8) | 386,204 (89.0) | – | 258,216 (57.8) | 433,093 (96.2) | – |
| Mean ± SD | 44.2 ± 14.5 | 36.7 ± 14.0 | 52.9* | 44.2 ± 14.5 | 42.8 ± 11.5 | 11.1* |
| Median (IQR) | 46.3 (33.3, 56.4) | 35.8 (24.3, 48.6) | – | 46.3 (33.3, 56.4) | 43.0 (37.0, 50.0) | – |
| ≥65 | 176,348 (39.5) | 0 (0.0) | – | 176,348 (39.5) | 0 (0.0) | – |
| Sex, *n* (%) |  |  |  |  |  |  |
| Female | 270,997 (60.7) | 258,374 (59.6) | 2.3 | 270,997 (60.7) | 281,703 (62.5) | 3.8 |
| Male | 175,371 (39.3) | 175,210 (40.4) | 2.3 | 175,371 (39.3) | 168,640 (37.4) | 3.8 |
| Other/unknown | 57 (0.0) | 172 (0.0) | 1.7 | 57 (0.0) | 56 (0.0) | 0.0 |
| Race, *n* (%) |  |  |  |  |  |  |
| White | 326,384 (73.1) | 324,415 (74.8) | 3.8 | 326,384 (73.1) | 321,800 (71.4) | 3.7 |
| Black or African American | 68,815 (15.4) | 51,529 (11.9) | 10.3* | 68,815 (15.4) | 73,766 (16.4) | 2.6 |
| Asian or Pacific Islander | 12,334 (2.8) | 15,246 (3.5) | 4.3 | 12,334 (2.8) | 12,986 (2.9) | 0.7 |
| Other/unknown^c^ | 38,892 (8.7) | 42,566 (9.8) | 3.8 | 38,892 (8.7) | 41,848 (9.3) | 2.0 |
| Ethnicity, *n* (%) |  |  |  |  |  |  |
| Hispanic or Latino | 44,226 (9.9) | 49,912 (11.5) | 5.2 | 44,226 (9.9) | 48,453 (10.8) | 2.8 |
| Non-Hispanic or Latino | 362,353 (81.2) | 349,468 (80.6) | 1.5 | 362,353 (81.2) | 360,532 (80.0) | 2.8 |
| Other/unknown | 39,846 (8.9) | 34,376 (7.9) | 3.6 | 39,846 (8.9) | 41,409 (9.2) | 0.9 |
| US geographic region, *n* (%) |  |  |  |  |  |  |
| West | 39,669 (8.9) | 28,754 (6.6) | 8.4 | 39,669 (8.9) | 41,143 (9.1) | 0.9 |
| Midwest | 190,013 (42.6) | 262,049 (60.4) | 36.3* | 190,013 (42.6) | 185,616 (41.2) | 2.7 |
| South | 87,799 (19.7) | 53,423 (12.3) | 20.2* | 87,799 (19.7) | 90,714 (20.1) | 1.2 |
| Northeast | 43,531 (9.8) | 36,741 (8.5) | 4.5 | 43,531 (9.8) | 43,850 (9.7) | 0.1 |
| Other/unknown | 85,413 (19.1) | 52,789 (12.2) | 19.3* | 85,413 (19.1) | 89,075 (19.8) | 1.6 |
| BMI, *n* (%)^d^ |  |  |  |  |  |  |
| <18.5 (underweight) | 2,101 (0.5) | 2,121 (0.5) | 0.3 | 2,101 (0.5) | 1,979 (0.4) | 0.5 |
| 18.5–24.9 (normal) | 42,635 (9.6) | 36,001 (8.3) | 4.4 | 42,635 (9.6) | 45,065 (10.0) | 1.5 |
| 25.0–29.9 (overweight) | 58,984 (13.2) | 35,044 (8.1) | 16.7* | 58,984 (13.2) | 64,589 (14.3) | 3.3 |
| ≥30 (obese) | 102,118 (22.9) | 0 (0.0) | – | 102,118 (22.9) | 0 (0.0) | – |
| Unknown | 240,587 (53.9) | 360,590 (83.1) | 66.3* | 240,587 (53.9) | 338,767 (75.2) | 45.7* |
| **Clinical characteristics** |  |  |  |  |  |  |
| EUA criteria, *n* (%)^e,f^ |  |  |  |  |  |  |
| Cardiovascular disease (including congenital heart disease) or hypertension | 222,663 (49.9) | 0 (0.0) | – | 222,663 (49.9) | 0 (0.0) | – |
| Age at index date ≥65 years | 176,348 (39.5) | 0 (0.0) | – |  |  | – |
| Immunosuppressive disease^g^ | 91,344 (20.5) | 0 (0.0) | – | 91,344 (20.5) | 0 (0.0) | – |
| Acquired heart disease | 105,634 (23.7) | 0 (0.0) | – | 105,634 (23.7) | 0 (0.0) | – |
| History of diabetes type 2 | 92,113 (20.6) | 0 (0.0) | – | 92,113 (20.6) | 0 (0.0) | – |
| Chronic lung disease (COPD or asthma) | 80,045 (17.9) | 0 (0.0) | – | 80,045 (17.9) | 0 (0.0) | – |
| Obesity^h^ | 123,833 (27.7) | 0 (0.0) | – | 123,833 (27.7) | 0 (0.0) | – |
| History of CKD (any stage) | 35,948 (8.1) | 0 (0.0) | – | 35,948 (8.1) | 0 (0.0) | – |
| Non-asthma and non-COPD chronic respiratory diseases | 35,402 (7.9) | 0 (0.0) | – | 35,402 (7.9) | 0 (0.0) | – |
| Heart failure | 27,212 (6.1) | 0 (0.0) | – | 27,212 (6.1) | 0 (0.0) | – |
| Liver disease | 27,007 (6.0) | 0 (0.0) | – | 27,007 (6.0) | 0 (0.0) | – |
| Pregnancy | 23,363 (5.2) | 0 (0.0) | – | 23,363 (5.2) | 0 (0.0) | – |
| History of CKD (stage ≥3) | 17,264 (3.9) | 0 (0.0) | – | 17,264 (3.9) | 0 (0.0) | – |
| Neurodevelopmental disorders | 23,684 (5.3) | 0 (0.0) | – | 23,684 (5.3) | 0 (0.0) | – |
| Pulmonary hypertension | 7,028 (1.6) | 0 (0.0) | – | 7,028 (1.6) | 0 (0.0) | – |
| Immunosuppressive treatment^i^ | 14,156 (3.2) | 0 (0.0) | – | 14,156 (3.2) | 0 (0.0) | – |
| Anti-diabetic therapies | 9,836 (2.2) | 0 (0.0) | – | 9,836 (2.2) | 0 (0.0) | – |
| History of diabetes type 1 | 5,547 (1.2) | 0 (0.0) | – | 5,547 (1.2) | 0 (0.0) | – |
| Acute or acute-on-chronic respiratory failure | 15,961 (3.6) | 0 (0.0) | – | 15,961 (3.6) | 0 (0.0) | – |
| A medical-related technological dependence^j^ | 5,140 (1.2) | 0 (0.0) | – | 5,140 (1.2) | 0 (0.0) | – |
| ICS-containing therapies | 7,777 (1.7) | 0 (0.0) | – | 7,777 (1.7) | 0 (0.0) | – |
| Sickle cell disease | 1,794 (0.4) | 0 (0.0) | – | 1,794 (0.4) | 0 (0.0) | – |
| Number of EUA criteria |  |  |  |  |  |  |
| Mean ± SD | 2.4 ± 1.9 | 0 ± 0.0 | – | 2.4 ± 1.9 | 0.0 ± 0.0 | – |
| Median (IQR) | 2.0 (1.0, 3.0) | 0.0 (0.0, 0.0) | – | 2.0 (1.0, 3.0) | 0.0 (0.0, 0.0) | – |
| Current or former smoker | 33,622 (7.5) | 2,751 (0.6) | 35.4* | 33,622 (7.5) | 4,967 (1.1) | 32.0 |
| CCI, mean (SD) | 0.9 ± 1.7 | 0.0 ± 0.2 | 77.5* | 0.9 ± 1.7 | 0.0 ± 0.3 | 76.3 |
| Concomitant medication, *n* (%) |  |  |  |  |  |  |
| Received any during baseline | 355,145 (79.6) | 200,046 (46.1) | 73.7* | 355,145 (79.6) | 241,794 (53.7) | 57.0 |
| Received any during acute phase | 187,646 (42.0) | 59,436 (13.7) | 66.6* | 187,646 (42.0) | 84,450 (18.7) | 52.3 |
| Healthcare utilization^k^ |  |  |  |  |  |  |
| Healthcare encounters during baseline^l^ |  |  |  |  |  |  |
| *n* (%) with any | 421,451 (94.4) | 355,327 (81.9) | 39.4* | 421,451 (94.4) | 364,700 (81.0) | 41.8* |
| Mean ± SD | 18.1 ± 21.5 | 6.1 ± 9.1 | 73.0* | 18.1 ± 21.5 | 6.5 ± 9.9 | 69.5* |
| Median (IQR) | 12.0 (5.0, 23.0) | 3.0 (1.0, 8.0) | – | 12.0 (5.0, 23.0) | 3.0 (1.0, 8.0) | – |
| Healthcare encounters during acute phase^l^ |  |  |  |  |  |  |
| *n* (%) with any | 307,407 (68.9) | 199,076 (45.9) | 47.7* | 307,407 (68.9) | 212,683 (47.2) | 44.9* |
| Mean ± SD | 2.4 ± 3.3 | 1 ± 1.7 | 53.6* | 2.4 ± 3.3 | 1.1 ± 1.9 | 48.7* |
| Median (IQR) | 1.0 (0.0, 3.0) | 0.0 (0.0, 1.0) | – | 1.0 (0.0, 3.0) | 0.0 (0.0, 1.0) | – |
| ICU encounters during acute phase |  |  |  |  |  |  |
| *n* (%) with any | 526 (0.1) | 34 (0.0) | 4.4 | 526 (0.1) | 60 (0.0) | 4.1 |
| Mean ± SD | 0.0 ± 0.0 | 0.0 ± 0.0 | 4.3 | 0.0 ± 0.0 | 0.0 ± 0.0 | 3.9 |
| Median (IQR) | 0.0 (0.0, 0.0) | 0.0 (0.0, 0.0) | – | 0.0 (0.0, 0.0) | 0.0 (0.0, 0.0) | – |
| **COVID-19 disease history** |  |  |  |  |  |  |
| Vaccination status, *n* (%)^m^ |  |  |  |  |  |  |
| Fully vaccinated with booster^n^ | 52,557 (11.8) | 54,626 (12.6) | 2.5 | 52,557 (11.8) | 56,228 (12.5) | 2.2 |
| Fully vaccinated without booster^o^ | 38,671 (8.7) | 21,857 (5.0) | 14.4* | 38,671 (8.7) | 31,609 (7.0) | 6.1 |
| Partially vaccinated^p^ | 9,487 (2.1) | 6,745 (1.6) | 4.2 | 9,487 (2.1) | 9,556 (2.1) | 0.0 |
| No record of vaccination | 345,710 (77.4) | 350,528 (80.8) | 8.3 | 345,710 (77.4) | 353,006 (78.4) | 2.3 |
| Time from last vaccination dose to index (months)^m^ | ***N = 100,715*** | ***N = 83,228*** |  | ***N = 100,715*** | ***N = 97,392*** |  |
| Mean ± SD | 7.6 ± 4.6 | 7.2 ± 4.7 | 9.0 | 7.6 ± 4.6 | 7.8 ± 5.6 | 4.3 |
| Median (IQR) | 7.3 (4.2, 9.9) | 7.0 (4.2, 9.2) | – | 7.3 (4.2, 9.9) | 7.0 (5.0, 10.0) | – |
| Test used to diagnose COVID-19, *n* (%) |  |  |  |  |  |  |
| PCR | 231,246 (51.8) | 280,672 (64.7) | 26.4* | 231,246 (51.8) | 232,728 (51.7) | 0.3 |
| Other/unknown^q^ | 215,179 (48.2) | 153,084 (35.3) | – | 215,179 (48.2) | 217,671 (48.3) | – |
| COVID-19 diagnosis in ER, *n* (%) | 73,788 (16.5) | 43,351 (10.0) | 19.4* | 73,788 (16.5) | 78,834 (17.5) | 2.6 |
| SARS-CoV-2 variant type based on time period of COVID-19 diagnosis, *n* (%)^r^ |  |  |  |  |  |  |
| Epsilon | 65,879 (14.8) | 69,189 (16.0) | 3.3 | 65,879 (14.8) | 66,145 (14.7) | 0.2 |
| Alpha | 24,032 (5.4) | 24,170 (5.6) | 0.8 | 24,032 (5.4) | 24,845 (5.5) | 0.6 |
| Delta | 61,779 (13.8) | 72,020 (16.6) | 7.7 | 61,779 (13.8) | 62,966 (14.0) | 0.4 |
| Omicron BA.1 (before Paxlovid^®^  availability)^s^ | 12,651 (2.8) | 16,726 (3.9) | 5.7 | 12,651 (2.8) | 12,973 (2.9) | 0.0 |
| Omicron BA.1 (after Paxlovid^®^  availability)^s^ | 89,921 (20.1) | 99,652 (23.0) | 6.9 | 89,921 (20.1) | 92,272 (20.5) | 0.9 |
| Omicron BA.2 | 102,261 (22.9) | 59,395 (13.7) | 24.0* | 102,261 (22.9) | 100,389 (22.3) | 1.5 |

*Abbreviations* ATT, average treatment effect in the treated; BMI, body mass index; CCI, Charlson comorbidity index; CKD, chronic kidney disease; COPD, chronic obstructive pulmonary disease; COVID-19, coronavirus disease 2019; CPT-4, Current Procedural Terminology; ER, emergency room; EUA, emergency use authorization; HCPCS, Healthcare Common Procedure Coding System; ICD-10, International Classification of Diseases, Tenth Revision; ICS, inhaled corticosteroid; ICU, intensive care unit; IQR, interquartile range; mRNA, messenger ribonucleic acid; N3C, National COVID Cohort Collaborative; PCR, polymerase chain reaction; SARS-CoV-2, severe acute respiratory syndrome coronavirus 2; SD, standard deviation; Std. diff., standardized difference; US, United States

*Indicates potential confounder (standardized difference >10%)

^a^Due to N3C database processing limitations, this analysis was conducted among a random sample of 25% of the eligible population

^b^ATT weights were calculated. Covariates included in the propensity score used to generate ATT weights were age, sex, race, ethnicity, US geographic region, BMI, vaccination status, vaccine types, PCR test for COVID-19 diagnosis, COVID-19 diagnosis in the ER, and SARS-CoV-2 variant type based on time period of COVID-19 diagnosis

^c^Other/unknown includes "Other race", "No information", "Unknown", "Multiple races", "West Indian", "Dominican Islander", and "Hispanic"

^d^Measurements that were negative or equal to zero were removed. Measurements were then trimmed at the bottom and top one percentile (1% and 99%) to reduce the influence of outliers. If BMI value was not directly available in the patient's measurement data, it was calculated as weight/(height)2*703, where weight is reported in pounds and height is reported in inches, and where 703 is the conversion factor from metric. The weight assessment during the baseline period and closest to the index date was considered for the analysis. If multiple assessments were available on the same day, the average value was used. The maximum value for height assessed during the baseline period was considered for the analysis

^e^Assessment period included the baseline period or index date for all criteria except age, which was assessed on the index date

^f^Patients may fit into more than one category; therefore, these categories are not mutually exclusive, and the percentages do not sum to 100%

^g^Immunosuppressive disease was defined based on ICD-10, CPT-4, and/or HCPCS codes relating to any of the following: Hodgkin's lymphoma, Non-Hodgkin's lymphoma, leukemia, solid cancers, HIV, autoimmune disease, solid organ transplant and/or allogenic stem cell transplant

^h^Obesity was defined based on laboratory measurements of BMI ≥30 or relevant ICD-10 codes

^i^Immunosuppressive treatment was defined based on HCPCS and/or NDC codes relating to systematic corticosteroid therapy or systemic non-corticosteroid immunosuppressants

^j^Medical-related technological dependence was defined based on ICD-10, CPT-4, and/or HCPCS codes relating to any of the following: respiratory aspirator, gastro- or jejunostomy, mitrofanoff, a nasogastric tube, renal replacement therapy, total parenteral nutrition, or ventricular assistance

^k^For patients with more than two visits on the same day (regardless of types of encounters), only one encounter was included

^l^Healthcare encounters includes inpatient visits (including ICU visits), outpatient visits, ER visits, and other types of visits such as long-term care visits and virtual visits

^m^Assessed during the vaccination assessment period which is defined as from the start of data availability until 14 days prior to the COVID-19 diagnosis date

^n^Defined as patients who received three or more vaccinations with an mRNA vaccine (i.e., Pfizer-BioNTech [BNT162b2] or Moderna [mRNA-1273]) or two or more vaccinations with at least one of those vaccinations being with the viral vector Johnson & Johnson vaccine (JNJ-784336725)

^o^Defined as patients who received two vaccinations with an mRNA vaccine (i.e., Pfizer-BioNTech [BNT162b2] or Moderna [mRNA-1273]) or one vaccination with the viral vector Johnson & Johnson vaccine (JNJ-784336725)

^p^Defined as patients who received one vaccine with an mRNA vaccine (Pfizer-BioNTech [BNT162b2] or Moderna [mRNA-1273])

^q^Other/unknown includes patients with unknown test types and patients whose first confirmed case of COVID-19 was determined by a rapid antigen test or clinical diagnosis

^r^The SARS-CoV-2 variant types were defined by the time of the patient's confirmed initial COVID-19 diagnosis period

^s^Omicron BA.1 time period was separated into two time periods before and after Paxlovid^®^ availability on December 21, 2021

**Supplementary Table S4** Comparative analysis of PASC in untreated high-risk versus untreated non-high-risk patients

|  | **Untreated high-risk**  **(*N* = 446,425)** | **Untreated non-high-risk**  **(*N* = 433,756)^a^**  ***(ATT weighted sample N = 450,399)*** | ***p*-value** |
| --- | --- | --- | --- |
| **PASC (main definition)^b^** |  |  |  |
| *N* (%) | 218,903 (49.0) | 108,706 (25.1) |  |
| Unweighted HR (95% CI) | 2.49 (2.47–2.51) | Reference | <0.001* |
| Doubly robust ATT-weighted HR (95% CI)^c^ | 2.08 (2.06–2.10) | Reference | <0.001* |
| **PASC (sensitivity definition)^d^** |  |  |  |
| *N* (%) | 4,510 (1.0) | 1,612 (0.4) |  |
| Unweighted HR (95% CI) | 2.77 (2.62–2.93) | Reference | <0.001* |
| Doubly robust ATT-weighted HR (95% CI)^c^ | 1.85 (1.72–1.99) | Reference | <0.001* |

*Abbreviations* ATT, average treatment effect in the treated; CI, confidence interval; COVID-19, coronavirus disease 2019; HR, hazard ratio; ICD-10, International Classification of Diseases, Tenth Revision; mAb, monoclonal antibody; N3C, National COVID Cohort Collaborative; PASC, post-acute sequelae of COVID-19; PCR, polymerase chain reaction; SARS-CoV-2, severe acute respiratory syndrome coronavirus 2

*Indicates statistical significance (*p* < 0.05)

^a^Due to data-processing limitations on the N3C database, this analysis was conducted among a random sample of 25% of the eligible population

^b^PASC was defined as demonstration of at least one of the 26 PASC symptoms (described in [2]) at least 30 days after the first ICD-10 diagnosis code for COVID-19 (U07.1) or positive PCR or antigen test in the observation period. The observation period was defined from the end of the acute phase until the earliest of day 365 following the diagnosis date, loss to follow-up, end of N3C data availability, death, COVID-19 re-infection (i.e., second positive SARS-CoV-2 PCR/antigen test at least 45 days after the first positive SARS-CoV-2 PCR/antigen test or diagnosis of COVID-19 and after at least one negative SARS-CoV-2 PCR/antigen test), or initiation of another mAb, antiviral, or pre-exposure prophylaxis

^c^The Cox regression model did not include further adjustment for any covariates as all characteristics were balanced with a standardized difference ≤10% after weighting

^d^PASC was defined based on: (1) the use of ICD-10 diagnosis code U09.9 on or after October 1, 2021 or the use of ICD-10 diagnosis code B94.8 prior to October 1, 2021 at least 30 days after the first ICD-10 diagnosis code for COVID-19 [U07.1] or positive PCR or antigen test; and/or (2) at least one visit to a PASC specialty clinic 30 days after first ICD-10 diagnosis code for COVID-19 [U07.1] or positive PCR or antigen test. The observation period is defined from the end of acute phase until the earliest of day 365 following the diagnosis date, loss to follow-up, end of N3C data availability, death, COVID-19 re-infection (defined as a second positive SARS-CoV-2 PCR/antigen test at least 45 days after the first positive SARS-CoV-2 PCR/antigen test or diagnosis of COVID-19 and after at least 1 negative SARS-CoV-2 PCR/antigen test), or initiation of another mAb, antiviral, or PrEP.

**Supplementary Table S5** Comparative analysis of PASC^a^ in untreated high-risk and untreated non-high-risk patients using multivariable Cox regression model^b,c^

|  | **HR (95% CI)** | ***p*-value** |
| --- | --- | --- |
| **Unadjusted** |  |  |
| Untreated high-risk versus untreated non-high-risk (reference) | 2.49 (2.47–2.51) | <0.001* |
| **Adjusted** |  |  |
| Untreated high-risk versus untreated non-high-risk (reference) | 2.23 (2.21–2.24) | <0.001* |
| Age at index date |  |  |
| <18 years | 0.90 (0.88–0.91) | <0.001* |
| ≥18 years | Reference |  |
| Sex |  |  |
| Female | Reference |  |
| Male | 0.88 (0.88–0.89) | <0.001* |
| Other/unknown | 0.53 (0.38–0.73) | <0.001* |
| Race |  |  |
| White | Reference |  |
| Black or African American | 1.04 (1.03–1.05) | <0.001* |
| Asian or Pacific Islander | 0.92 (0.90–0.94) | <0.001* |
| Other/unknown^d^ | 0.88 (0.87–0.89) | <0.001* |
| Ethnicity |  |  |
| Non-Hispanic or Latino | Reference |  |
| Hispanic or Latino | 0.95 (0.94–0.96) | <0.001* |
| Other/unknown | 1.05 (1.04–1.07) | <0.001* |
| US geographic region |  |  |
| West | Reference |  |
| Midwest | 0.79 (0.78–0.80) | <0.001* |
| South | 0.99 (0.98–1.01) | 0.23 |
| Northeast | 0.72 (0.71–0.74) | <0.001* |
| Other/unknown | 1.06 (1.04–1.07) | <0.001* |
| BMI^e^ |  |  |
| <18.5 (underweight) | 1.04 (0.99–1.09) | 0.14 |
| 18.5–24.9 (normal) | Reference |  |
| 25.0–29.9 (overweight) | 1.03 (1.01–1.04) | <0.001* |
| Smoking status during baseline |  |  |
| Non-smoker | Reference |  |
| Current or former smoker | 1.48 (1.46–1.50) | <0.001* |
| Type of test used to diagnose COVID-19 |  |  |
| Other/unknown test | Reference |  |
| PCR test | 0.96 (0.95–0.97) | <0.001* |
| SARS-CoV-2 variant type based on time period of  COVID-19 diagnosis^f^ |  |  |
| Delta | 0.95 (0.94–0.96) | <0.001* |
| Omicron BA.1 (before Paxlovid^®^ availability)^g^ | 0.97 (0.95–0.99) | <0.001* |
| Omicron BA.1 (after Paxlovid^®^ availability)^g^ | Reference |  |
| Omicron BA.2 | 1.04 (1.03–1.05) | <0.001* |
| Setting of COVID-19 diagnosis |  |  |
| Diagnosis of COVID-19 in other settings | Reference |  |
| Diagnosis of COVID-19 in the emergency room | 1.17 (1.16–1.18) | <0.001* |

*Abbreviations* ATT, average treatment effect in the treated; BMI, body mass index; CI, confidence interval; COVID-19, coronavirus disease 2019; HR, hazard ratio; ICD-10, International Classification of Diseases, Tenth Revision; mAb, monoclonal antibody; N3C, National COVID Cohort Collaborative; PASC, post-acute sequelae of COVID-19; PCR, polymerase chain reaction; SARS-CoV-2, severe acute respiratory syndrome coronavirus 2; US, United States

*Indicates statistical significance (*p* < 0.05)

^a^PASC was defined as demonstration of at least one of the 26 PASC symptoms (described in [2]) at least 30 days after the first ICD-10 diagnosis code for COVID-19 (U07.1) or positive PCR or antigen test in the observation period. The observation period was defined from the end of the acute phase until the earliest of day 365 following the diagnosis date, loss to follow-up, end of N3C data availability, death, COVID-19 re-infection (i.e., second positive SARS-CoV-2 PCR/antigen test at least 45 days after the first positive SARS-CoV-2 PCR/antigen test or diagnosis of COVID-19 and after at least one negative SARS-CoV-2 PCR/antigen test), or initiation of another mAb, antiviral, or pre-exposure prophylaxis

^b^The multivariable Cox regression model adjusted for covariates included in the propensity score used to generate ATT weights and the Cox regression model from the main analysis, which included age, sex, race, ethnicity, US geographic region, BMI, current or former smoker during baseline, PCR test for COVID-19 diagnosis, COVID-19 diagnosis in the emergency room, and SARS-CoV-2 variant type based on the time period of COVID-19 diagnosis

^c^Out of 446,425 patients in the untreated high-risk group, 218,903 (49.0%) had PASC in the observation period. Out of 433,756 patients in the untreated non-high-risk group, 108,706 (25.1%) had PASC in the observation period

^d^Other/unknown includes "Other race", "No information", "Unknown", "Multiple races", "West Indian", "Dominican Islander", and "Hispanic"

^e^Measurements that were negative or equal to zero were removed. Measurements were then trimmed at the bottom and top one percentile (1% and 99%) to reduce the influence of outliers. If BMI value was not directly available in the patient's measurement data, it was calculated as weight/(height)2*703, where weight is reported in pounds and height is reported in inches, and where 703 is the conversion factor from metric. The weight assessment during the baseline period and closest to the index date was considered for the analysis. If multiple assessments were available on the same day, the average value was used. The maximum value for height assessed during the baseline period was considered for the analysis

^f^The SARS-CoV-2 variant types were defined by the time of the patient's confirmed initial COVID-19 diagnosis period

^g^Omicron BA.1 time period was separated into two time periods before and after Paxlovid^®^ availability on December 21, 2021

## Supplementary Fig. S1 Cohort disposition (30-day acute phase). (a) Sotrovimab-treated and untreated high-risk patients, and (b) untreated high-risk and untreated non-high-risk patients

(a)


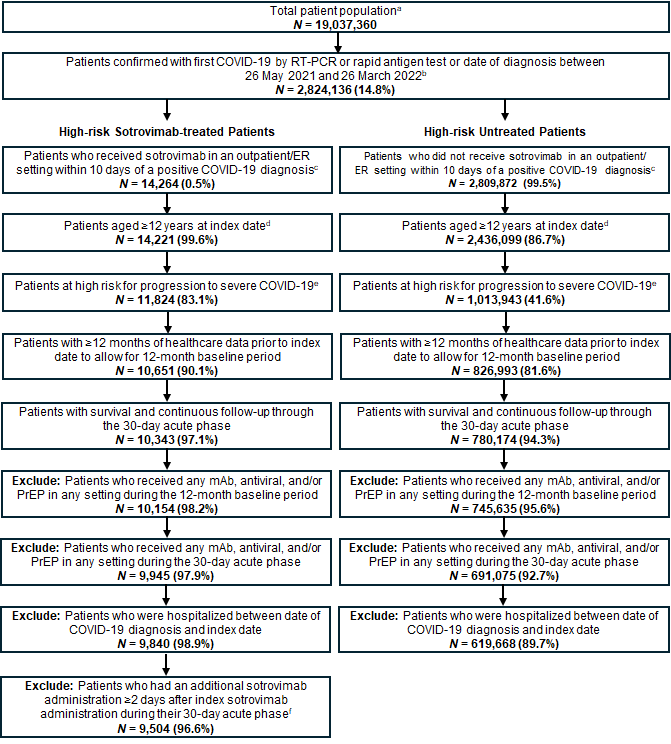


(b)


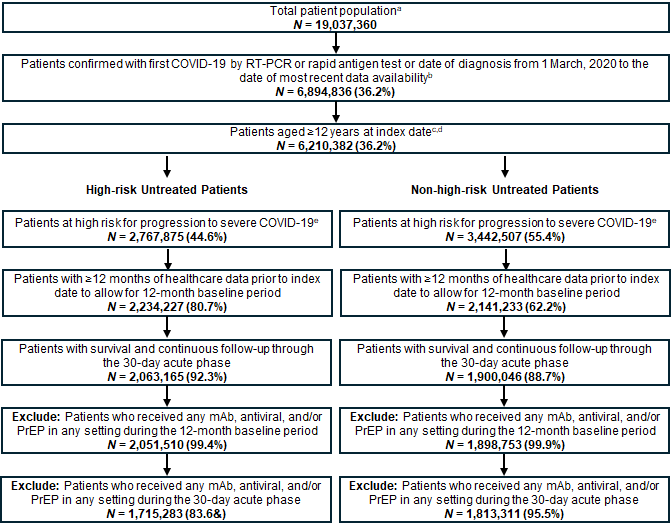


*Abbreviations* COVID-19, coronavirus disease 2019; ER, emergency room; mAb, monoclonal antibody; PrEP, pre-exposure prophylaxis; RT-PCR, reverse transcription polymerase chain reaction

^a^Patients were identified from the NCATS National COVID Cohort Collaborative (N3C), a centralized, secure clinical data resource created by the National Institute of Health. N3C is a continually updated database, and the data shown here are based on N3C data downloaded on 06/15/2023.
^b^COVID positive diagnoses were determined from lab and condition data by using the phenotype algorithm identified by N3C: https://github.com/National-COVID-Cohort-Collaborative/Phenotype_Data_Acquisition/wiki/Latest-Phenotype."
^c^For patients who received sotrovimab within 10 days from the date of a positive COVID-19 diagnosis, the first sotrovimab administration identified between 26 May 2021 (i.e., EUA issued by FDA for sotrovimab) and 05 April 2022 (i.e., EUA reversal by the FDA) was assigned as the index date. To avoid immortal time bias, index dates were imputed for patients who did not receive sotrovimab. Index dates were imputed using the distribution for number of days from a positive COVID diagnosis to the administration date of sotrovimab among sotrovimab users. These index dates were generated at this step since they were needed for the application of the remaining inclusion/exclusion criteria.
^d^Patients were further excluded if they have missing birth date or were older than 100 years at their index date.
^e^Patients fulfilling any one or more of the EUA criteria, at index date, are classified as being at high risk of developing severe COVID-19.
^f^An additional sotrovimab administration received <2 days after index sotrovimab administration may be due to data entry errors or heterogenous prescription practices across hospitals. Patients with such records are considered to have only received their index sotrovimab administration during the acute phase and were therefore included in the sample. Patients who had an additional sotrovimab administration ≥2 days after their index sotrovimab administration during the acute phase were excluded to ensure index sotrovimab is the only treatment received during the acute phase.

**References**

1. Bowe B, Xie Y, Xu E, Al-Aly Z. Kidney outcomes in long COVID. J Am Soc Nephrol. 2021;32:2851–62.

2. Bull-Otterson L, Baca S, Saydah S, Boehmer TK, Adjei S, Gray S, et al. Post–COVID conditions among adult COVID-19 survivors aged 18–64 and ≥65 years — United States, March 2020–November 2021. MMWR Morb Mortal Wkly Rep. 2022;71:713–17.

3. Daugherty SE, Guo Y, Heath K, Dasmariñas MC, Jubilo KG, Samranvedhya J, et al. Risk of clinical sequelae after the acute phase of SARS-CoV-2 infection: retrospective cohort study. BMJ. 2021;373:n1098.

4. Sharafeldin N, Bates B, Song Q, Madhira V, Yan Y, Dong S, et al. Outcomes of COVID-19 in patients with cancer: report from the National COVID Cohort Collaborative (N3C). J Clin Oncol. 2021;39: 2232–46.

5. Taquet M, Dercon Q, Luciano S, Geddes JR, Husain M, Harrison PJ. Incidence, co-occurrence, and evolution of long-COVID features: a 6-month retrospective cohort study of 273,618 survivors of COVID-19. PLoS Med. 2021;18:e1003773.

6. Taquet M, Geddes JR, Husain M, Luciano S, Harrison PJ. 6-month neurological and psychiatric outcomes in 236 379 survivors of COVID-19: a retrospective cohort study using electronic health records. Lancet Psychiatry. 2021;8:416–27.

7. Xie Y, Bowe B, Al-Aly Z. Burdens of post-acute sequelae of COVID-19 by severity of acute infection, demographics and health status. Nat Commun. 2021;12:6571.

8. Xie Y, Xu E, Al-Aly Z. Risks of mental health outcomes in people with covid-19: cohort study. BMJ. 2022;376:e068993.

9. Xie Y, Xu E, Bowe B, Al-Aly Z. Long-term cardiovascular outcomes of COVID-19. Nat Med. 2022;28:583–90.

10. Pfaff ER, Girvin AT, Bennett TD, Bhatia A, Brooks PM, Deer RR, et al. Who has long-COVID? A big data approach. medRxiv. 2021:2021.10.18.21265168.

11. Pfaff ER, Madlock-Brown C, Baratta JM, Bhatia A, Davis H, Girvin A, et al. Coding long COVID: characterizing a new disease through an ICD-10 lens. BMC Med. 2023;21:58.

12. Rando HM, Bennett TD, Byrd JB, Bramante C, Callahan TJ, Chute CG, et al. Challenges in defining Long COVID: striking differences across literature, Electronic Health Records, and patient-reported information. medRxiv. 2021:2021.03.20.21253896.

13. Reese JT, Blau H, Casiraghi E, Bergquist T, Loomba JJ, Callahan TJ, et al. Generalisable long COVID subtypes: findings from the NIH N3C and RECOVER programmes. EBioMedicine. 2023;87:104413.

14. Davis HE, Assaf GS, McCorkell L, Wei H, Low RJ, Re'em Y, et al. Characterizing long COVID in an international cohort: 7 months of symptoms and their impact. EClinicalMedicine. 2021;38:101019.

15. Centers for Disease Control and Prevention. Long COVID or post-COVID conditions. 2024. https://www.cdc.gov/coronavirus/2019-ncov/long-term-effects/index.html. Accessed June 30, 2024.

16. World Health Organization. Statement on Omicron sublineage BA.2. 2022. https://www.who.int/news/item/22-02-2022-statement-on-omicron-sublineage-ba.2. Accessed June 30, 2024.

17. Al-Aly Z, Bowe B, Xie Y. Long COVID after breakthrough SARS-CoV-2 infection. Nat Med. 2022;28:1461–7.

18. Al-Aly Z, Xie Y, Bowe B. High-dimensional characterization of post-acute sequelae of COVID-19. Nature. 2021;594:259–64.

19. Carvalho-Schneider C, Laurent E, Lemaignen A, Beaufils E, Bourbao-Tournois C, Laribi S, et al. Follow-up of adults with noncritical COVID-19 two months after symptom onset. Clin Microbiol Infect. 2021;27:258–63.

20. Galván-Tejada CE, Herrera-García CF, Godina-González S, Villagrana-Bañuelos KE, Amaro JDL, Herrera-García K, et al. Persistence of COVID-19 symptoms after recovery in Mexican population. Int J Environ Res Public Health. 2020;17:9367.

21. Carfì A, Bernabei R, Landi F; Gemelli Against COVID-19 Post-Acute Care Study Group. Persistent symptoms in patients after acute COVID-19. JAMA. 2020;324:603–5.

22. Chopra V, Flanders SA, O'Malley M, Malani AN, Prescott HC. Sixty-day outcomes among patients hospitalized with COVID-19. Ann Intern Med. 2021;174:576–8.

23. Sonnweber T, Boehm A, Sahanic S, Pizzini A, Aichner M, Sonnweber B, et al. Persisting alterations of iron homeostasis in COVID-19 are associated with non-resolving lung pathologies and poor patients' performance: a prospective observational cohort study. Respir Res. 2020;21:276.

24. Soriano JB, Murthy S, Marshall JC, Relan P, Diaz JV; WHO Clinical Case Definition Working Group on Post-COVID-19 Condition. A clinical case definition of post-COVID-19 condition by a Delphi consensus. Lancet Infect Dis. 2022;22:e102–7.

25. Pfaff ER, Girvin AT, Bennett TD, Bhatia A, Brooks IM, Deer RR, et al; N3C Consortium. Identifying who has long COVID in the USA: a machine learning approach using N3C data. Lancet Digit Health. 2022;4:e532–41.
